# Strain belonging to an emerging, virulent sublineage of ST131 *Escherichia coli* isolated in fresh spinach, suggesting that ST131 may be transmissible through agricultural products

**DOI:** 10.3389/fcimb.2023.1237725

**Published:** 2023-10-09

**Authors:** Maria G. Balbuena-Alonso, Manel Camps, Gerardo Cortés-Cortés, Eder A. Carreón-León, Patricia Lozano-Zarain, Rosa del Carmen Rocha-Gracia

**Affiliations:** ^1^ Posgrado en Microbiología, Centro de Investigaciones Microbiológicas, Instituto de Ciencias, Benemérita Universidad Autónoma de Puebla, Puebla, Mexico; ^2^ Departament of Microbiology and Environmental Toxicology, University of California at Santa Cruz, Santa Cruz, CA, United States; ^3^ Facultad de Ciencias Químicas, Universidad Autónoma de Chihuahua, Chihuahua, Mexico

**Keywords:** ExPEC, ST131, food safety, virulence, mobile genetic elements, conjugative transfer

## Abstract

Food contamination with pathogenic *Escherichia coli* can cause severe disease. Here, we report the isolation of a multidrug resistant strain (A23EC) from fresh spinach. A23EC belongs to subclade C2 of ST131, a virulent clone of Extraintestinal Pathogenic *E. coli* (ExPEC). Most A23EC virulence factors are concentrated in three pathogenicity islands. These include PapGII, a fimbrial tip adhesin linked to increased virulence, and *CsgA* and *CsgB*, two adhesins known to facilitate spinach leaf colonization. A23EC also bears Tn*MB1860*, a chromosomally-integrated transposon with the demonstrated potential to facilitate the evolution of carbapenem resistance among non-carbapenemase-producing enterobacterales. This transposon consists of two IS*26*-bound modular translocatable units (TUs). The first TU carries *aac(6’)-lb-cr*, *bla*_OXA-1_, *ΔcatB3*, *aac(3)-lle*, and *tmrB*, and the second one harbors *bla*_CXT-M-15_. A23EC also bears a self-transmissible plasmid that can mediate conjugation at 20°C and that has a mosaic IncF [F(31,36):A(4,20):B1] and Col156 origin of replication. Comparing A23EC to 86 additional complete ST131 sequences, A23EC forms a monophyletic cluster with 17 other strains that share the following four genomic traits: (1) virotype E (*papGII*+); (2) presence of a PAI II_536_-like pathogenicity island with an additional *cnf1* gene; (3) presence of chromosomal Tn*MB1860*; and (4) frequent presence of an F(31,36):A(4,20):B1 plasmid. Sequences belonging to this cluster (which we named “C2b sublineage”) are highly enriched in septicemia samples and their associated genetic markers align with recent reports of an emerging, virulent sublineage of the C2 subclade, suggesting significant pathogenic potential. This is the first report of a ST131 strain belonging to subclade C2 contaminating green leafy vegetables. The detection of this uropathogenic clone in fresh food is alarming. This work suggests that ST131 continues to evolve, gaining selective advantages and new routes of transmission. This highlights the pressing need for rigorous epidemiological surveillance of ExPEC in vegetables with One Health perspective.

## 1 Introduction

Food products are a key source for transmission of pathogenic bacteria, such *as Salmonella* and *Escherichia coli*. (Gizaw, 2019) *E. coli* colonizes the human gastrointestinal tract as a commensal but some pathotypes of *E. coli* can cause serious disease (Denamur et al., 2021). Pathogenic strains of *E. coli* can be grouped into intestinal (InPEC) and extraintestinal (ExPEC) pathotypes depending on whether they cause primarily gastrointestinal symptoms or whether they affect other organs, including urinary tract, bloodstream, wounds, kidney, brain, and other internal organs (Sora et al., 2021). ExPEC strains are responsible for a large fraction of urinary tract and bloodstream infections in humans (Sarowska et al., 2019) and can be classified into 4 pathotypes: uropathogenic *E. coli* (UPEC, primarily associated with urinary tract infections-UTIs), avian pathogenic *E. coli* (APEC, which causes colibacillosis in poultry but that can also infect humans), septicemia-associated *E. coli* (SEPEC) and neonatal meningitis-causing *E. coli* (NMEC) (Meena et al., 2023).

Community and hospital ExPEC infections are generally treated with 2^nd^ and 3^rd^ generation cephalosporins, fluoroquinolones, or with trimethoprim-sulfamethoxazole (Pitout, 2012). Resistance to cephalosporins can generally be attributed to the acquisition of genes with extended-spectrum β-lactamase (ESBL) activity; in ExPEC, the most frequent ones are *bla*_CTX-M_ variants. Resistance to fluoroquinolones is largely due to mutations that reduce the affinity of these drugs for their targets (gyrase -*gyrAB*- and topoisomerase IV -*parCE-*), although resistance can also be provided or enhanced by a fluoroquinolone-degrading enzyme (*aac(6’)-lb-cr*), by DNA-protecting enzymes (*qnr* genes), or by efflux regulation mutations (*marR*, *acrR* and *soxR*) (Huseby et al., 2017; Poirel et al., 2018). Resistance to dihydrofolate metabolism inhibitors is generally provided by dihydrofolate synthetase genes that are insensitive to sulfonamides (*sul*) or by dihydrofolate reductase mutants (*dfr*) that are insensitive to trimethoprim (Van Duijkeren et al., 2000). Topoisomerase genes represent core functions and are most frequently found in the chromosome. The other resistance genes are accessory and tend to be found in plasmids, which in turn facilitate their spread, mostly by conjugation. Over time, plasmid-borne genes tend to get incorporated into chromosomes with the assistance of mobile genetic elements (MGEs) such as insertion sequences and transposons (Wang Y. et al., 2022).

Sequence type 131 (ST131) is a globally-dominant multidrug resistance (MDR) clone and a major driver of the current ExPEC pandemic. Compared to other globally dispersed clones, ST131 stands out for its highly dynamic accessory genome (Decano et al., 2021). Like most UPEC strains, ST131 resides in the human gut as a commensal but can cause mild to severe infections of urinary tract and asymptomatic urinary bacteriuria (Biggel et al., 2020; Tchesnokova et al., 2020). The ST131’s genome exhibits a broad range of known or suspected virulence factors that likely contributed to its dominance in the clinic. These include siderophores, adhesins, toxins, protectins and other elements contributing to the successful establishment and persistence of an infection. Some of these factors are specific to ST131 sublineages, notably *hly* (hemolysin), *iro* (siderophore), AAF (aggregative adherence fimbriae), and *papGII* (P fimbrial tip adhesin variant).

The complex population structure of ST131 has been elucidated (Price et al., 2013; Petty et al., 2014). Two ancestral clades have been identified, namely clade A (which carries the *fimH41* allele) and clade B (which carries the *fimH22* and *fimH35* allelic variants). Strains belonging to these two clades, which are now infrequent, are generally sensitive to fluoroquinolones and rarely carry ESBL plasmids, although they occasionally have *bla*_CTX-M_ genes integrated in the chromosome. Clade C is an emerging epidemic lineage characterized by multidrug resistance and higher virulence (Denamur et al., 2021). This clade appears to have evolved from clade B by the acquisition of the *fimH30* allele and of several prophages and it expresses serotype O25b:H4. Within clade C, three subclades can be distinguished by divergent antimicrobial resistance profiles: C0, which is sensitive to fluoroquinolones, and C1 and C2, which have acquired fluoroquinolone resistance mutations in *gyrA* and *parC*. C1 and C2 in turn, differ in the *bla*_CTX-M_ gene associated with them: subset C1 (with *fimH30*R1), is typically associated with *bla*_CTX-M-14_ or with *bla*_CTX-M-27_, whereas subset C2 (with *fimH30*RX), is generally associated with *bla*_CTX-M-15_. The *bla*_CTX-M-15_ gene was initially carried by IncF plasmids but more recently the mobilization of this gene to the chromosome has been reported through transposition events frequently involving insertion sequences belonging to the IS*26* family (Dhanji et al., 2011; Stoesser et al., 2016). The C2 subclade has been found to be enriched in highly virulent and multidrug resistance isolates, with the virulence associated with the presence of the gene *papGII* (Pajand et al., 2021; Biggel et al., 2022).

ST131’s main reservoir is thought to be the human intestine and the usual route of transmission to be person-to-person contact (Lopez et al., 2014; Pitout and Finn, 2020); however, an increasing (although still small) number of studies are reporting its isolation from non-human sources, such as food, food animals, pets, and environmental sources, suggesting that these may represent vectors for the transmission of this strain (Meena et al., 2023). ST131 strains recovered from food have been associated primarily with meat products, particularly pork and chicken (Meena et al., 2021); because of ST131’s ability for long-time residence in the gastrointestinal tract, animal carcasses contaminated with intestinal content are considered the likely source in meat products. It also needs to be noted that, in addition to contaminating food, *E. coli* food strains can also function as reservoirs of virulence and antibiotic resistance genes, which can be frequently exchanged with clinical strains *via* horizontal gene transfer (HGT) (Balbuena-Alonso et al., 2022).

Here we report the isolation, phenotypic characterization, and genomic sequence of an *E. coli* isolate from a spinach sample that we named A23EC. We describe the presence of a large number of virulence factors, mainly located in three pathogenicity islands, that suggest a uropathogenic pathotype. Also, we show that this strain presents a multidrug resistance profile that is consistent with its antibiotic resistance gene (ARG) content, and we describe a conjugative plasmid that is capable of self-transmission even at room temperature. Finally, we compare A23EC’s genomic sequence with that of other fully-assembled ST131 genomes in *GenBank* and find that A23EC belongs to a virulent, emerging sublineage of the ST131 subclade C2. We identify genetic markers unique to this sublineage and report on their alignment with earlier studies of virulent sublineages of the C2 subclade that were based on partially assembled sequences. These results highlight the pressing need for rigorous epidemiological surveillance of ExPEC in vegetables with a One Health perspective.

## 2 Materials and methods

### 2.1 Produce fresh sampling and bacterial isolation

During the period from April 2017 to November 2018, ready-to-eat raw vegetables were collected from fixed and mobile food service establishments, in Puebla, Mexico (19.03 N, -98.20 W). A total of 183 samples were obtained from 82 establishments with lettuce (21%), tomato (17%), onion with coriander (13%), cucumber (12%), onion (9%), carrot (6%), radish (6%), coriander (5%), brussels sprouts (3%), red onion (3%), celery (1%), spinach (1%), a mix of tomato, onion and coriander (1%), squash (1%), and green bell pepper (1%). All samples were stored at 4°C and processed within 24 h. For bacterial isolation, 10g of food were inoculated in 20 mL of sodium lauryl sulfate broth (BD Bioxon ®) and incubated at 37°C with shaking at 30 rpm for 24 h. Subsequently, the cultures were streaked onto MacConkey agar plates (BD Bioxon) supplemented with cefotaxime (CTX) (2ug/ml). Colonies were picked from the selective plates, subcultured and streaked to obtain pure cultures. Putative strains were identified according to biochemical tests with Vitek system (bioMérieux, France) following the schemes in MacFaddin’s Manual of Biochemical Tests for the Identification of Clinically Important Bacteria (MacFaddin, 2003). Also, *E. coli*-specific *ybbW* and *uidA* genes were used to confirm their identity by PCR (Silkie et al., 2008; Walker et al., 2017).

### 2.2 Antimicrobial susceptibility testing and detection of resistance genes

The antibiotic susceptibility profile was obtained by agar dilution methods using criteria from Clinical and Laboratory Standards Institute (CLSI) guidelines as a reference (CLSI, 2022); as a control, the strain *E. coli* ATCC 25922 was used. Twenty-one antimicrobial agents belonging to 12 antibiotic classes were tested: amikacin (30 ug), gentamicin (10 ug), streptomycin (10 ug), tobramycin (10 ug), ampicillin (10 ug), amoxicillin/clavulanic acid (20/10 ug), cefuroxime (30 ug), cefotaxime (30 ug), ceftazidime (30 ug), cefepime (30 ug), cefoxitin (30 ug), aztreonam (30 ug), trimethoprim (5 ug), trimethoprim/sulfamethoxazole (1.25/23.75 ug), nalidixic acid (30 ug), ciprofloxacin (5 ug), chloramphenicol (30 ug), tetracycline (30 ug), meropenem (10 ug), imipenem (10 ug) and fosfomycin/G6P (200/50 ug) (BBLTM Sensi-DiscTM; Becton Dickinson and Co). Cefotaxime resistant (CTX-resistant) *E. coli* isolates were screened for ESBL production by the double-disk synergy test using disks containing cefotaxime, ceftazidime, aztreonam and cefepime, with a centrally positioned disk of amoxicillin/clavulanic acid (20/10 ug).

### 2.3 DNA isolation and whole-genome sequencing analysis

The ESBL-producing *E. coli* strain isolated from spinach was selected for whole-genome sequencing (WGS). This strain (A23EC) was cultured overnight at 37°C in brain heart infusion broth; total DNA extraction was performed using the Wizard® genomic DNA purification kit (Promega, United States) according to the manufacturer’s instruction. Sequencing was perform using two platforms: shorts reads were generated on the Illumina Nextseq 500 plataform using 75-bp paired-end (Ilumina, United States) and long reads were generated on the Nanopore Minion plataform depth 80x (Nanopore, United, States). Quality check of the raw sequencing data was performed using Quast v0.11.5 (https://www.bioinformatics.babraham.ac.uk/projects/fastqc/) (Wingett and Andrews, 2018). In the next step, hybrid genome assembly was generated with SPAdes v3.9.0 and Unicycler v0.5.0 assemblers (Bankevich et al., 2012; Wick et al., 2017). Finally, annotation was performed by the Rapid Annotations using Subsystems Technology (RAST) server (https://rast.nmpdr.org/rast.cgi (accessed on June 17, 2022) (Aziz et al., 2008) and using PROKKA v1.2 (Seemann, 2014). Sequence type was characterized using MLST v2.0 (https://pubmlst.org/organisms/escherichia-spp (accessed on June 25, 2022), and pMLST (http://pubmlst.org/plasmid/) (Villa et al., 2010); the serotypes (O:H) were predict with SerotypeFinder v2.0 (Joensen et al., 2015) whereas phylogroup was determined using the ClermonTyping 1.4 (Clermont et al., 2013) (http://clermontyping.iame-research.center), and finally the variant of the *fimH* gene was determined using FimTyper v1.0 (https://cge.cbs.dtu.dk/services/FimTyper) (Roer et al., 2017).

### 2.4 Plasmid analysis

PFGE with S1 nuclease (S1 Nuclease Thermo Scientific) digestion of whole genomic DNA was used for determinate the number and size of plasmids of strain A23EC (Ben Sallem et al., 2014). PlasmidFinder 2.1 (Carattoli et al., 2014) (https://cge.cbs.dtu.dk/services/PlasmidFinder/) (85% identity and 70% minimum length) was used to identify the replicon present for each plasmid and the replicase type. The presence of conjugation elements in plasmids was performed by using the tool OriTfinder 1.1 (Li et al., 2018) (https://bioinfo-mml.sjtu.edu.cn/oriTfinder/) with modificated parameters (Blast E-value 0.00001) and the identity of relaxase was confirmed with MOBScan (Garcillán-Barcia et al., 2020) (https://castillo.dicom.unican.es/mobscan). Also, Plasmid Taxonomic Units (PTU) were identified by COPLA (a taxonomic classifier of plasmids) using the recommended parameters (Redondo-Salvo et al., 2020) (https://castillo.dicom.unican.es/copla/). The search for type I (*pndAD, srnBC* and *hok-sok*) and type II (*ccdAB, relEB, parDE, pemKI* and *vagCD*) addiction systems was performed by BLASTn alignment with the parameters 90% coverage, 80% identity and E-value ≤ 0.000001; the system was considered to be present if the Antitoxin-Toxin (AT) sequences were adjacent. Finally, Blast Ring Image Generator (BRIG) v0.95 was used with default settings to compare our plasmid sequences to publicly available sequence (Alikhan et al., 2011) and visualized using Proksee.ca 1.0 (Stothard et al., 2018).

### 2.5 Detection of virulence and resistance genes

Antimicrobial resistance genes were detected using ResFinder v4.1 (https://cge.cbs.dtu.dk/services/ResFinder/)(Bortolaia et al., 2020) (with parameters 80% of coverage and 90% of identity) and the Comprehensive Antibiotic Resistance Database (CARD) v3.2.5 (https://card.mcmaster.ca/analyze) (McArthur et al., 2013) by the Resistance Gene Identifier (RGI) with the “strict” algorithm. The virulence gene profile of the isolate was established based on the detection of these genes in VirulenceFinder v2.0 (the cut-off values for genes identity was 90% and alignment coverage was 70%) (https://cge.cbs.dtu.dk/services/VirulenceFinder/) (Tetzschner et al., 2020), Virulence Factor database (VFDB) and VFanalyzer with default parameters (http://www.mgc.ac.cn/cgi-bin/VFs/v5/main.cgi, accessed on October 20, 2022) (Liu et al., 2019). The virotype was assigned according to the presence of virulence genes following the scheme described by Nicholas-Chanoine et al, and the additional ones suggested by Barrios-Villa et al. (Nicolas-Chanoine et al., 2014; Barrios-Villa et al., 2018). The Pathogenicity Islands were predicted using IslandViewer 4, which uses three independent methods for island prediction: IslandPick, IslandPath-DIMOB and SIGI-HMM (https://www.pathogenomics.sfu.ca/islandviewer)(Bertelli et al., 2017), sequence comparison of each island was performed using Blastn v2.13.and Easyfig v2.2.5 (Sullivan et al., 2011) using the sequences of the prototype strains reported by Desvaux et al., 2020. Finally, PHASTER web server was used to predict prophage regions in the genome of A23EC strain (https://phaster.ca/) (Arndt et al., 2016). Insertion sequences and transposons were identified with Mobile Element Finder v1.0.3 (https://cge.food.dtu.dk/services/MobileElementFinder/) (Johansson et al., 2021) and the identity of the insertion sequences was confirmed using ISfinder (Siguier et al., 2006); while VRprofile2 (https://tool2-mml.sjtu.edu.cn/VRprofile/) (Wang M. et al., 2022) was used to identify the genetic environment of the resistance genes. CD-HIT-EST (Li and Godzik, 2006) was used to cluster similar structures with more than 99% coverage and similarity, and a multiple genetic structural comparison was represented using Easyfig v2.2.5 (Sullivan et al., 2011).

### 2.6 Phylogenetic analysis

We compared the *E. coli* strain A23EC with other 86 fully assembled *E. coli* ST131 genomes deposited in *GenBank*, belonging to clades A, B and C. The metadata collected was the source of isolation, year of sampling, and country of origin (**Table S1**). All the *E. coli* genomes referred to as “Clinical” (66 strains) were associated with human infections recovered from urine, blood, sputum, and feces. The category “Animal” (4 strains) represented samples of dog, cat, and pig. The category “Environmental” (3 strains) includes strains isolated from wastewater bodies. Thirteen strains were not classified according to their sample type, and they were marked as “No data available”. The unweighted Pair-Group Method using Arithmetic averages (UPGMA) was performed based on SNPs, using the newick graph obtained with CSI Phylogeny (https://cge.cbs.dtu.dk/services/CSIPhylogeny-1.2/, accessed on June 27, 2022) (Kaas et al., 2014) with EC598 as reference genome (accession number NZ_HG941718.1) (Forde et al., 2014). The SNP tree was visualized using iTOL software (https://itol.embl.de) (Letunic and Bork, 2021).

### 2.7 Conjugation assays

The two strains : donor (A23EC^Tet+^) and recipient (C600^Rif+^): donor (A23EC^Tet+^) and recipient (C600^Rif+^) were cultured separately in 5mL Luria Bertani broth (LB) and incubated at 37°C overnight. Subsequently, 1000 μL of the recipient and 250 μL of the donor were mixed and inoculated into 20 mL of LB, incubated for 24 hours at 37°C and 20°C with shaking at 130 rpm. Finally, serial dilutions from 100 to 10-6 or 1,000,000 were performed and plated for selection of transconjugants (Cortés-Cortés et al., 2016). Transconjugants were selected using MacConkey agar (BD Bioxon) supplemented with rifampicin (100μg/mL) and tetracycline (10μg/mL). To confirm the identity of the transconjugants, PCR amplifications of the *tetA* (plasmid location) and *bla*_CTX-M-15_ (chromosomal location) genes were performed. Only *tetA* was expected to be amplified in the transconjugants. Each experiment was performed in triplicate. The plasmid transference under the two temperatures was compared by *t-student* in Rstudio statistical software 1.4.1103. The frequency of conjugation (Fc) is described in this study as the ratio of the number of transconjugants divided by the number of recipients (Huisman et al., 2022; Mota-Bravo et al., 2023), expressed by the following equation:


$$FC=\frac{Transconjugants/\left(\frac{CFU}{mL}\right)}{Recipient/\left(\frac{CFU}{mL}\right)}$$

### 2.8 Accession numbers

*GenBank* accession numbers of the strain A23EC genome sequenced for this study are Chromosome CP118558.1 and plasmid pA23EC CP118559.1 Bioproject: PRJNA936840. BioSample: SAMN33377322.

## 3 Results

### 3.1 Isolation of an ExPEC strain from spinach

As part of a study involving 183 vegetable samples, in 2017-2018 we isolated a CTX-resistant strain from a spinach sample obtained in a supermarket in the city of Puebla, Mexico. The spinach sample came from a bulk-salad section that was not contained in sealed packaging. The sample was collected into an individual sterile bag, stored at 4°C and transported immediately to the laboratory for processing. Biochemical tests identified the isolate as being *E. coli* and this identity was confirmed using conventional PCR amplification of two diagnostic markers for *E. coli* (*ybbW* and *uidA*, not shown).

Next, we obtained the complete genomic sequence of this strain, which we named A23EC, using the Illumina Nextseq 500 and Nanopore Minion platforms, generating a hybrid assembly using short and long sequences, respectively. According to its sequence, A23EC belongs to phylogroup B2, sequence type ST131 and serotype O25:H4. The presence of allelic variant *fimH30Rx* identifies this strain as belonging to clade C, subclade C2, a lineage that includes highly virulent clones (Stoesser et al., 2016; Pajand et al., 2021; Biggel et al., 2022). The closest relative to A23EC in *GenBank* is the p4A strain (CP049085.2), isolated from a patient suffering from bacteremia in the United States in 2015 (**Table S2**) and reported in Shropshire et al., 2021. The genome features, virulome and resistome of *E. coli* A23EC strain are summarized in **Table 1**.

**Table 1:** Genome features, virulome and resistome of *E. coli* A23EC strain.

Feature	Chromosome	Plasmid pA23EC
Size (bp)	5,239,797	157,470
GC (%)	50.6	50.3
No. of genes	5253	217
Inc group (pMLST)	NA	FII : FIA:FIB : Col156
Virulome		
Virulence genes	*csgAB, iss, sitA, malX, yehABCD, yfcV, chuA, fimABCDFGH, usp, ompT*	*senB, iutA, traT, iucD*
Pathogenicity island (PAI)	CFT073 (I, II), 536 (II)	
Resistome		
Aminoglycosides	*aac(3)-IIe, aac(6’)-lb-cr*	
Betalactams	*bla*_CTX-M-15_, *bla*_OXA-1_	
Quinolones	*aac(6’)-lb-cr*	
QRDR quinolones	*gyrA* (S83L, D87N), *parC* (S80I)	
Macrolides	*mdfA*	
Phenicols	Δ*catB3*	
Tetracyclines		*tetAR*
Metals	*baeRS, cpxA, pmrF*	
Biocides	*acrABDFS, evgAS, mdtBCEFGHMNOP, sitA, marA, emrABEKRY, tolC, gadWX*	
Heat stress tolerance	*KpnEF*	

NA, not applicable; QRDR, quinolone resistance-determining region.

**Figure 1:**
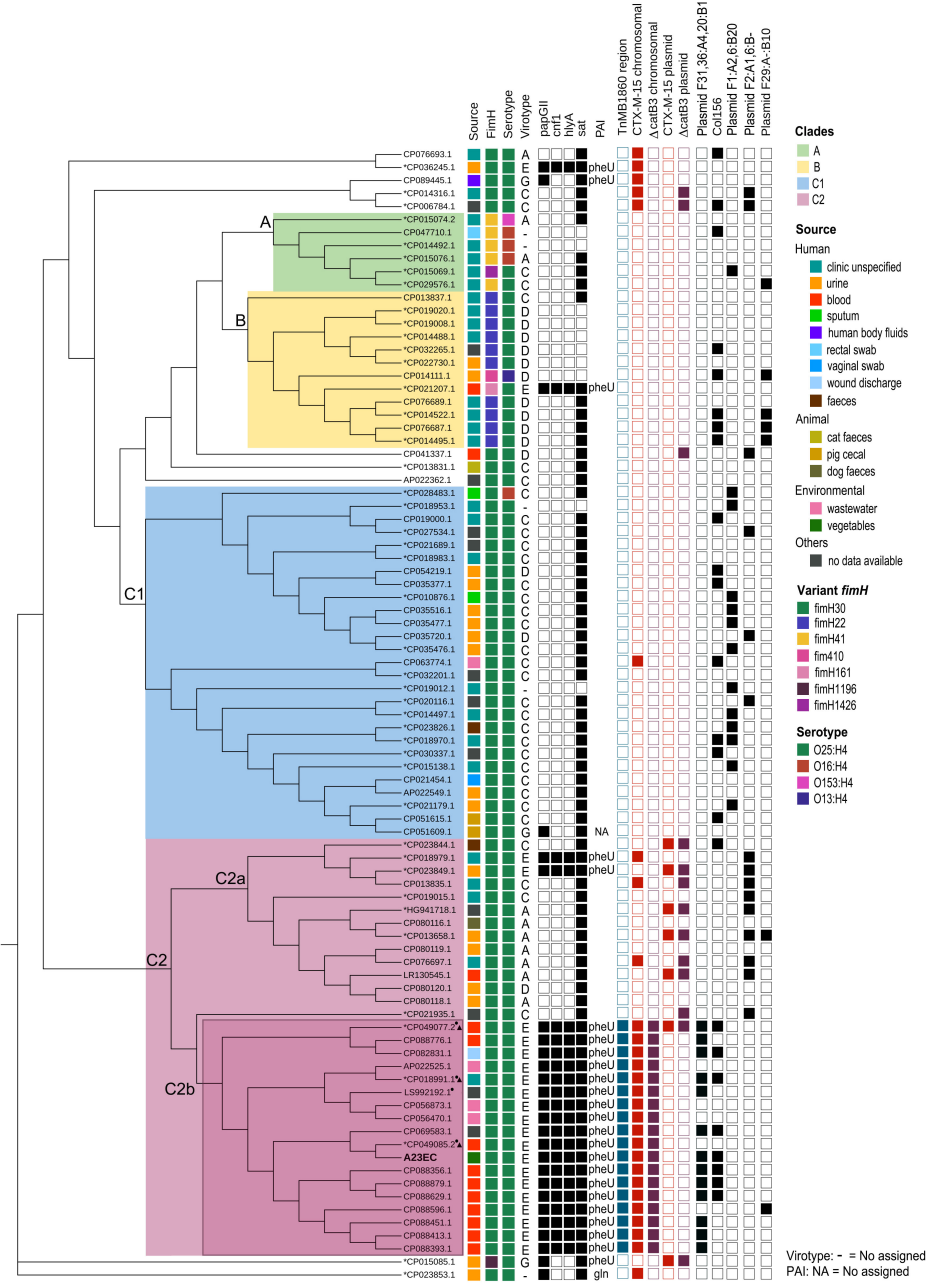
Distribution of selected genetic elements across ST131 sublineages. Cladogram showing the phylogenetic relationships between the 87 genomes included in this study. The first column indicates the source of isolation. The second column indicates the allelic variant of FimH identified. The third column indicates the serotype. The fourth column shows lists the virotype assigned. The 5th to 8th columns indicate the virulence genes that define virotype E. The 9th column indicates the tRNA insertion site of the pathogenicity island harbouring *cnf1, papGII* and *hly* genes. The 10th shows the presence of the compound transposon Tn*MB1860*. Columns 11-14 show the presence of Δ*catB3* and CTX-M-15 and whether they are found in a plasmid or in the chromosome). The 15th column indicates the presence of plasmid (F31,36:A4,20:B1). Next column shows presence of the Col156 replicon. The last three columns indicate the presence the other frequent F plasmid replicons (those with a frequency of occurrence > 5 plasmids) listed according to their pMLST classification. The asterisk next to the *GenBank* accession number indicates strains contained in the study of Biggel et al., 2022 and those with a small triangle belong to the L1 sublineage. The circle indicates strains included in the study of Shropshire et al., 2021. This graphical representation was generated using the CSI Phylogeny platform with EC598 (NZ_HG941718.1) as the reference genome.

To see how A23EC relates to other ST131 strains, we performed a phylogenetic analysis of A23EC along with all other complete ST131 genomes of *E. coli* deposited in *GenBank* until June 2022. The results, shown in the form of a cladogram are presented in **Figure 1**. The 86 ST131 genomes included in this analysis group into the three known ST131 clades, namely A, B, and C. Within the C clade, they group into two main subclades (C1 and C2). Within the C2 subclade, we see two distinct monophyletic clusters (sublineages), that we named C2a (13 strains) and C2b (18 strains). A23EC belongs to sublineage C2b. **Figure 1** also lists the source of the samples (when known). Genomes corresponding to sublineage C2b largely correspond to human clinical strains, with three environmental strains isolated from wastewater. The geographic origin of sublineage C2b samples is listed in **Table S1**, and includes samples from Europe, Asia, Australia and North and South America.

### 3.2 Pathogenicity molecular profile

**Figure 2:**
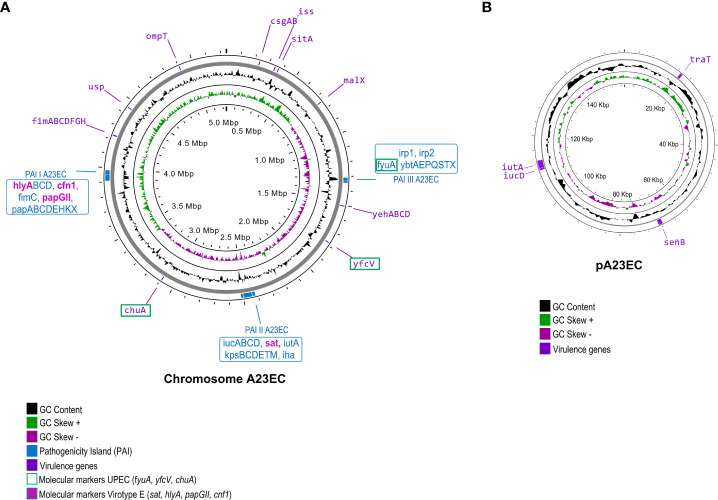
Virulome of *E. coli* A23EC. The genome is shown, with GC content, + and – skew in the inner circles. The outer circle indicates the location of individual virulence genes, labelled in their genomic positions. The labels corresponding to virulence factors serving as molecular markers of uropathogenic strains are boxed in green. **(A)** Chromosomal virulome. The three PAI I pathogenicity islands identified in A23EC are shown. PAI I A23EC (similar to PAI-II-536-pheU [PAI II_536_]), PAI II A23EC (similar to PAI-CFT073-pheV [PAI I_CFT073_]), and PAI III A23EC (similar to PAI-CFT073-asnT [PAI II_CFT073_] and corresponding to pathogenicity island [HPI]) (Llyod et al., 2009). The virulence genes harbored in each PAIs are listed in blue boxes. Virotype E-defining genes, all found within these PAIs, are highlighted in bold and pink. **(B)** Plasmid (pA23EC) virulome. Genes encoding virulence factors are labeled and their position is shown in purple boxes.

Using IslandViewer 4, we identified three pathogenicity islands, mapped to the chromosome in **Figure 2**. The first one was PAI I A23EC (this PAI is similar to PAI II_536_), which contained the genes *hlyABCD* (hemolysin encoding cluster), *fimC (*chaperone-like periplasmic protein*)* and *papABCDEHKX* and *papGII* (P-fimbrial tip adhesin). Unlike PAI II_536_, PAI I_A23EC_ also carries *cnf1* (cytotoxic necrotizing factor). The second pathogenicity island is PAI II A23EC (which is similar to PAI I_CFT073_), which harbored the *iucABCD* genes, members of a family of non-ribosomal peptide synthetase-independent siderophore (NIS), *sat* (secreted autotransporter toxin), *kpsMII-K5* (capsular protein variant K5), *iha* (iron-regulated gene homologue adhesin), and *iutA* (ferric aerobactin receptor). Finally, the third pathogenicity island is PAI III A23EC. This PAI is similar to PAI II_CFT073_ corresponding to high pathogenicity island (HPI) (Schubert et al., 2004; Llyod et al., 2009) including the siderophore yersiniabactin biosynthesis and uptake genes *irp1, irp2, fyuA* and *ybtAEPQSTX* (Perry et al., 1999).

Virulencefinder identified twenty-four additional virulence genes in the genome of A23EC. These genes can be grouped into the following five functional categories: 1) bacterial adhesion (*csgAB, yfcV* and operon *fimABCDFGHI*); 2) iron acquisition (*sitA, chuA, iucD and iutA*); 3) serum resistance (*iss*, and *traT*); 4) colonization and invasion (*ompT, yehABCD* and *malX*); and 5) toxin genes (*usp* and *senB*) (**Figures 2**, **S1**).

### 3.3 Phenotypic and genotypic antibiotic resistance profile

A23EC’s antibiotic resistance profile and ESBL status was determined using the Kirby-Bauer method. Phenotypically, A23EC is a MDR strain (Magiorakos et al., 2012), exhibiting resistance to at least one member of six different antibiotic families: aminoglycosides, penicillin, cephalosporins, tetracyclines, quinolones and monobactams. In addition, our A23EC strain showed a positive ESBL phenotype and intermediate resistance profile for amikacin and amoxicillin with clavulanic acid. The only antibiotics tested that this strain remained susceptible to were carbapenems, fosfomycin, chloramphenicol and trimethoprim with sulfamethoxazole (**Table S3**).

Using Resfinder and RGI CARD, we found the following ARGs (**Table 1**): two genes encoding β-lactamase (*bla*_CTX-M-15_ and *bla*_OXA-1_), one aminoglycoside resistance gene (*aac(3)-IIe*) and an aminoglycoside and quinolone resistance gene (*aac(6’)-lb-cr*), an efflux pump (*mdfA*), a truncated chloramphenicol resistance gene (Δ*catB3*), a tunicamycin resistance determinant (*tmrB*) (Noda et al., 1992), and the gene *tetA* associated with resistance to tetracycline (alongside *tetR*, transcriptional repressor). The truncated Δ*catB3* gene harbors two in-frame deletions involving a total of 28 amino-acids (see nucleotide alignment in **Figure S3**). These two deletions reduce the size of this 210 amino-acid protein to 182 amino acids, and result in a loss of homology with respect to the WT beginning at amino acid position 147 (see amino acid alignment in **Figure S3B**), thus deleting the entire C-terminal α-helical domain.

The *gyrA* subunit of gyrase and the *parC* subunit of topoisomerase IV had three point mutations conferring fluoroquinolone resistance: S83I, D87N in gyrase and S80L in topoisomerase IV. The presence of *bla*_CTX-M-15_ and of fluoroquinolone resistance mutations in *gyrA* and *parC* are consistent with the placement of this strain in subclade C2 of ST131.

### 3.4 Genomic structure of chromosomal ARGs

**Figure 3:**
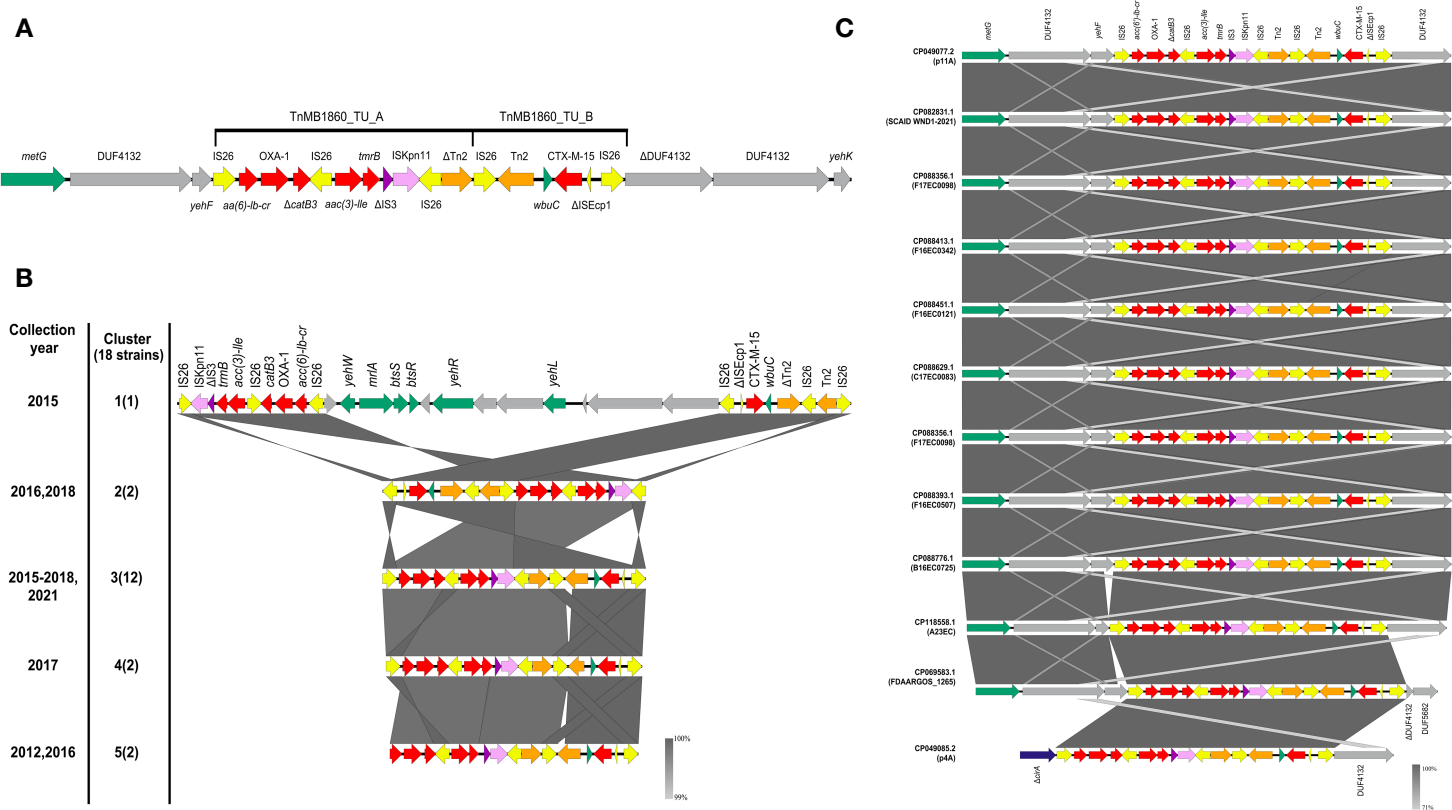
Genomic structure and genetic context of the Tn*MB1860* sequence in the C2b sublineage of the C2 subclade of ST131 genomes included in our study. The different genetic elements are shown as arrows. Resistance genes are shown in red; IS26 insertion sequences in yellow, ΔIS3 in purple, IS*Kpn11* in lilac, transposons in orange, *metG* in green, Δ*cirA* in blue and genes encoding hypothetical proteins in gray. Genes within and in the vicinity of Tn*MB1860* are labelled. **(A)** Structure of Tn*MB1860*. The genetic structure of Tn*MB1860* with its two transposable units and a total of six ARGs, as described in Shropshire et al, 2021 (Shropshire et al., 2021) **(B)** Comparative genetic arrangement of Tn*MB1860* in genomes belonging to sublineage C2b. The five clusters reflect distinct genetic distribution profiles of Tn*MB1860*. The *GenBank* accession numbers for the genomic sequences included in each group are shown in **Table S2**. The numbers on the left indicate the cluster corresponding to the set of sequences shown on the right, with number of sequences in each cluster shown in parentheses. All sequences were chromosomal, except for the single sequence in Cluster 1. The comparison was performed with Easyfig and the BLASTn algorithm. The percent sequence identity is shown in the form of a heatmap ranging between 99 and 100%, as shown in the legend. **(C)** Comparative genetic arrangement of the sequences in cluster 3. In eleven of the sequences, including strain A23EC, the insertion site of Tn*MB1860* was close to the gene encoding methionyl-tRNA synthetase, *metG*. A single sequence (CP049085.1) was instead inserted in the gene encoding for the truncated colicin I receptor (*cirA*), as previously described by Shropshire et al., 2021 (Shropshire et al., 2021).

Looking at the distribution of ARG genes in the chromosome, we found Tn*MB1860*, a 12,837 bp- *IS26*-bounded transposon structure integrated in the chromosome, previously described by Shropshire et al, 2021. Tn*MB1860* is made up of two discrete, modular translocatable units (TUs): MB1860TU_A and MB1860TU_B (**Figure 3A**). MB1860TU_A carries the *aac(6’)-lb-cr*, *bla*_OXA-1_ and truncated Δ*catB3* genes flanked by two IS*26* elements in opposite orientations, followed by two additional antibiotic resistance genes (*aac(3)-lle* and *tmrB*) and is bordered on the 3’ end by a ΔIS*3*, IS*Kpn11*, IS*26* and ΔTn*2* cluster. MB1860TU_B is bounded by two IS*26* elements in the sense orientation and it has a partial IS*Ecp* sequence, a *bla*_CXT-M-15_ gene and the *wbuC* gene (also known as *orf477*) in the opposite orientation; its genetic organization is common for *bla*_CTX-M-15_ (Dhanji et al., 2011; Ludden et al., 2020).

Chromosomal TnMB1860 was previously reported in two non-carbapenemase producing septicemia isolates by Shropshire et al., 2021 (Shropshire et al., 2021), that (like A23EC) belonged to subclade C2 of ST131. To establish the distribution of Tn*MB1860* (flanked by IS*26*) more widely, we looked for the presence of the complete sequence in 2,387complete genomes of *E. coli*. As a threshold, we selected 98% sequence identity with a coverage >70%, then further confirmed that it was a similar arrangement by detecting the presence of the six relevant resistance genes within a 20,000 bp window. Based on these criteria, we found 18 highly significant hits, all of them chromosomal except for one (**Table S2**). Remarkably, all the 17 chromosomal sequences belong to the C2b sublineage of subclade C2 identified by our phylogenetic analysis shown in **Figure 1**. This observation suggests that Tn*MB1860* was captured during the evolution of the C2 subclade C2b and passed down vertically, making it a good identifier for sublineage C2b (**Figure 1**). In sixteen of these sequences (including that of strain A23EC) the insertion site of Tn*MB1860* is found ~4,400 bp away from the gene encoding methionyl-tRNA synthetase, *metG*, in a molybdopterin cofactor biosynthesis operon. In only one strain (p4A; sequence CP049085.1) Tn*MB186* was located adjacent to the gene encoding colicin I receptor, disrupting it (Δ*cirA*) (**Figure 3C**). The consistency between insertion sites and the observed clustering of this transposon’s representation in the cladogram (which points to vertical transmission) suggests that this is likely the result of a single capture event. The one exception in strain p4A presumably appears to be the result of an additional IS*26*-mediated intramolecular transposition event so it is still consistent with the hypothesis of a single capture event (Shropshire et al., 2021).

We also found a single Tn*MB1860* sequence located in a plasmid. This plasmid was p11A_p2, a 180,962 bp plasmid described by Shropshire et al, 2021. Plasmid p11A_p2 includes the MB1860TU_A and MB1860TU_B TUs located downstream of the class I integron carrying *dfrA17*, *aadA5*, *qacE*Δ1 and *sul1*. The two Tn*MB1860* TUs are not contiguous, though, but separated by a large insertion sequence (18,314 bp in length) containing 9 ORFs that include several virulence factors (**Figure 3B**). Plasmid p11A_p2 was found in strain 11A (CP049077.2), which also contained Tn*MB1860* integrated in the chromosome (Shropshire et al., 2021).

A comparative analysis of the structure of the Tn*MB1860* region in the sequences included in the C2b sublineage resulted in five distinct clusters (clusters #1 to 5). The canonical gene arrangements for each cluster are shown in **Figures 3B, C** and **S4**. The majority of genomic sequences (n=12) belong to cluster 3; all strains in this cluster show an identical structural profile of Tn*MB1860* (delimited by IS*26*); as mentioned above, strain p4A (CP049085.2) differs from the remaining eleven, including strain A23EC, in its insertion site (Δ*cirA* **),** but it shows an otherwise identical structure (**Figure 3C**). Cluster 1 contains the single plasmid Tn*MB1860* sequence (described above). Cluster 2, with two sequences, has MB1860TU_B translocated just upstream of MB1860TU_A in an inverted orientation, as well as an inversion of the IS*26* elements flanking the composite transposon (**Figure 3B**). Cluster 4, with two sequences, exhibits a loss of Tn*2* (**Figure 3C**). Finally, cluster 5, also with two sequences, exhibits a loss of IS*26* flanking the 5’ end of the MB1860TU_A region, as well as a Tn*2* element (**Figure 3B**).

### 3.5 Identification and characterization of plasmid content

Whole genome sequencing identified a single plasmid in the A23EC genome, which we named pA23EC. The presence of this plasmid was confirmed using the S1-PFGE technique (Cortés-Cortés et al., 2016). This plasmid is 157,470 bp in size, has a GC content of 50.3% and contains 172 putative coding regions (CDS) according to Prokka and RAST. Of these, only two CDSs (*tetA* and *tetR*) corresponded to ARGs, both part of a tetracycline resistance operon. These two genes were located contiguously and flanked by Tn*As1*. VirulenceFinder and VFanalyzer identified four virulence genes in pA23EC, namely *iutA, iucD* (related to iron uptake), *traT* (associated with serum resistance) and *senB* (encoding enterotoxin) (**Figure 2**).

**Figure 4:**
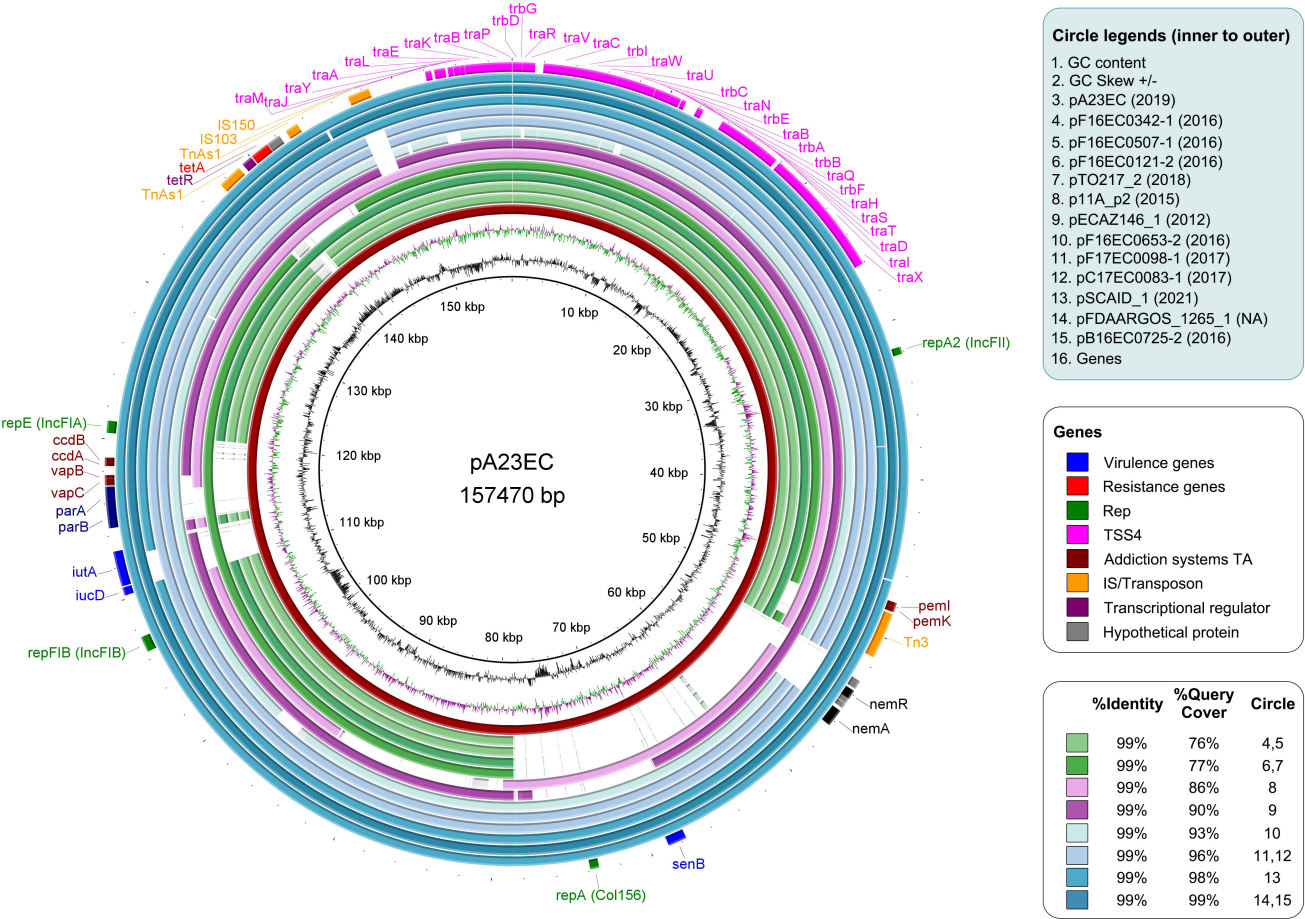
Comparative analysis of plasmid pA23EC with the other 11 F subtype FII_31/36:FIA_4/20:B1 plasmids identified within the sublineage C2b of subclade C2. From inner to outer relative to the distance map, the circles represent the following: Circle 1-2: GC content and GC Skew. Circle 3: plasmid pA23EC (highlighted in red). Circles 4-15: other plasmids belonging to subtype FII(31/36):FIA (4/20):B1 carried by the C2b sublineage of the C2 subclade of *E. coli* ST131; the names of the plasmids are listed in the legend, with their year of isolation in parenthesis. The % identity and % coverage are color-coded as indicated in the legend and shown in increasing coverage order. Circle 16: pA23EC gene content annotated with Prokka, with virulence, resistance, replication, conjugation, addiction systems, insertion sequences/transposons, transcription factors and hypothetical proteins in different colors, as indicated in the legend.

In terms of plasmid regulatory elements, PlasmidFinder identified four replicons, namely Col156, IncFII, IncFIA, and IncFIB. The three IncF replicons are widely separated from each other in the plasmid sequence and their alleles correspond to the F(31,36):A(4,20):B1 subclassification proposed by Villa et al. (Villa et al., 2010). The Col156 replicon is separated from the closest IncF replicon by 30,748 bp. COPLA, which is the most accurate method available for plasmid classification according to phylogenetic relatedness (Redondo-Salvo et al., 2021), ascribed pA23EC to the FE plasmid taxonomic unit (PTU-FE). We also found an abundance of plasmid maintenance systems, these including three type II toxin-antitoxin systems (*ccdAB, vapBC* and *pemKI*) and the *parAB* genes coding for a partitioning system that ensures accurate plasmid segregation (**Figure 4**, outer circle).

We looked for the presence of additional F(31,36):A(4,20):B1 plasmids in the 86 genomes shown in **Figure 1** and found eleven other examples, all within the C2 subclade C2b sublineage that includes strain A23EC. These plasmids showed a 99% percentage of identity to pA23EC, although the coverage (*i.e.* the degree of overlap) varied between 76% and 99%. A comparison of the 12 pA23EC-like plasmids identified is shown in **Figure 4**. The % coverage and elements that are absent in other plasmids relative to pA23EC are shown in **Table 2**. We see that two areas tend to get lost as the % overlap decreases. The first area is located between 50 kb and 80 kb and includes the genes *nemR, nemA* (involved in protection against oxidative stress) (Gray et al., 2013), the virulence gene *senB* and the Col156 replicase *repA*. The second area that is prone to deletion is located between positions 110 and 120 kb and includes the iron acquisition genes *iucD, iutA*, the partitioning system *parAB* and *vapBC*, and the addiction systems *ccdAB*. Tetracycline resistance is only lost in one of the plasmids (pTO217_2).

**Table 2:** Genetic elements that are absent relative to pA23EC; pA23EC-related plasmids are listed in decreasing order of overlap.

Plasmid	% query cover	*RepA*	*tetAR*	*nemR*	*nemA*	*senB*	*iucD*	*iutA*	*parAB*	*vapBC*	*pemIK*	*ccdAB*	Tn3	IS150
pA23EC	100	**+**	**+**	**+**	**+**	**+**	**+**	**+**	**+**	**+**	**+**	**+**	**+**	**+**
pB16EC0725-2	99	**+**	**+**	**+**	**+**	**+**	**+**	**+**	**+**	**+**	**+**	**+**	**+**	**+**
pFDAARGOS_1265_1	99	**+**	**+**	**+**	**+**	**+**	**+**	**+**	**+**	**+**	**+**	**+**	**+**	**+**
pSCAID_1	98	**+**	**+**	**+**	**+**	**+**	**+**	**+**	**+**	**+**	**+**	**+**	**+**	**+**
pF17EC0083-1	96	**+**	**+**	**-**	**-**	**+**	**+**	**+**	**+**	**+**	**+**	**+**	**+**	**-**
pF17EC0098-1	96	**+**	**+**	**-**	**-**	**+**	**+**	**+**	**+**	**+**	**+**	**+**	**+**	**-**
pF16EC0653-2	93	**+**	**+**	**-**	**-**	**+**	**+**	**+**	**+**	**+**	**+**	**+**	**+**	**-**
pECAZ146	90	**-**	**+**	**+**	**+**	**-**	**+**	**+**	**-**	**-**	**+**	**+**	**+**	**-**
p11A_p2	86	**+**	**+**	**-**	**+**	**+**	**-**	**-**	**-**	**+**	**+**	**+**	**+**	**+**
pTO217_2	77	**-**	**-**	**-**	**-**	**-**	**+**	**+**	**+**	**+**	**-**	**+**	**-**	**+**
pF16EC0121-2	77	**-**	**+**	**-**	**-**	**-**	**-**	**-**	**-**	**-**	**+**	**-**	**+**	**+**
pF16EC0507-1	76	**-**	**+**	**-**	**-**	**-**	**-**	**-**	**-**	**-**	**+**	**-**	**+**	**+**
pF16EC0342-1	76	**-**	**+**	**-**	**-**	**-**	**-**	**-**	**-**	**-**	**+**	**-**	**+**	**+**

### 3.6 Mobilization properties

In terms of propagation, the plasmid pA23EC was classified as self-transmissible (conjugative) based on the presence of a complete set of mobilization genes, which includes an *oriT* sequence, the type F relaxase protein (MOBF) and the type IV secretion system (TSS4) (Smillie et al., 2010). The type IV coupling protein (T4CP) showed intact domains (Garcillán-Barcia et al., 2020). We tested the conjugation ability of pA23EC at two temperatures, 20°C (the approximate temperature in the field) and 37°C (body temperature). As a recipient, we used a standard recipient strain C600^Rif+^. We did detect a low frequency of conjugation and this frequency did not differ significantly depending on the temperature, with a frequency of 2.65 × 10^−5^ at 37°C and a frequency of 2.82 × 10^−5^ at 20°C (*t-student*, p>0.05).

## 4 Discussion

Here we describe the isolation of a strain (A23EC) from a spinach sample at point-of-sale in Mexico and present a detailed genomic characterization of this isolate. Based on its sequence, this strain belongs to the C2 subclade of ST131, which is recognized as a pandemic clone that is highly virulent, multidrug resistant, and widely distributed around the world (Petty et al., 2014).

A phylogenetic analysis of all complete genomes corresponding to ST131 *E. coli* deposited in the *Genbank* database as of June 2022, places A23EC in a monophyletic sublineage of subclade C2, which we named C2b, clustered with seventeen other strains. These 18 genomes showed four unique commonalities, illustrated in **Figure 1**: 1) They carry a Tn*MB1860* transposon structure flanked by IS*26* elements. This transposon is chromosomally integrated in all cases and its chromosomal integration site in these genomes is consistent with a single capture event. 2) They carry a PAI II536-like pathogenicity island with an additional *cnf1* gene. 3) They consistently belong to virotype E. 4) Thirteen of the eighteen isolates (including A23EC) also carry a F(31,36):A(4,20):B1 plasmid. We did not detect this pMLST type in any other ST131 sample included in our analysis, suggesting that it is specific for sublineage C2b samples.

By contrast, strains belonging to the C2a sublineage included in the analysis lack the Tn*MB1860* transposon and frequently carry *bla*_CTX-M-15_ in plasmids, belong mostly to virotypes A or C (only two out of thirteen belong to virotype E); and consistently have F2:A-:B- plasmids when plasmids are present.

Note that all strains belonging to sublineage C2a were collected before 2014 (mean isolation date, 2009), whereas 16 of the 18 strains belonging to sublineage C2b were collected after 2014 (mean collection date, 2017), suggesting that C2b represents an emerging sublineage of the C2 subclade; this could be confirmed by further analysis with a larger number of strains.

Recent studies of ST131 clade C clinical strain collections already point to the emergence of new lineages of subclade C2 exhibiting higher virulence and antibiotic resistance. These virulent lineages are consistent with C2b, although in these studies, the identification of genomic markers was less comprehensive because most of the sequences were not completely assembled. Specifically, one study looked into the association of the gene *papGII* with the expansion of ST131 (Biggel et al., 2022). This report noted a large expansion of *papGII*+ isolates within the C2 subclade and distinguished three *papGII*+ sublineages: L1, L2 and L3. We noticed that the three fully sequenced strains from that study correspond to sublineage C2b in our analysis (marked with a triangle next to the accession number in **Figure 1**) and carry all the genetic markers that we defined as diagnostic for C2b. In that study, these strains were ascribed to L1 sublineage of *papGII*+ C2 strains (along with 233 additional samples). The L1 sublineage was defined by the dominant presence of F(31,36):A(4,20):B1 alleles in IncF plasmids, and by the dominant presence of *bla*_CTX-M-15_ as ESBL-encoding gene; further, the sub-branch they were ascribed to, L1a, consisting of 184 sequences is (like C2b) characterized by the insertion of Tn*MB1860* in the vicinity of the *metG* gene and by the presence of the two virulence genes *hly*/*cnf1* in the *papGII+* PAI. Thus, our proposed C2b sublineage appears to align perfectly with the L1a sub-branch of the Biggle et al., 2022 study (Biggel et al., 2022). They found two additional distinct *papGII+* sublineages within the C2 subclade. Given that these additional sublineages (L2 and L3) were less frequent and more geographically restricted (Ludden et al., 2020; Biggel et al., 2022), their absence in our study can likely be attributable to our much smaller sample size.

A second study identified a monophyletic cluster of 22 C2 strains enriched for virulence and for ARG markers that was named by the authors “C2 subset” (Pajand et al., 2021). Similar to our proposed C2b sublineage, this C2 subset was characterized by the presence of *hlyABCD* virulence genes found in a PAI II_536_-like genomic island containing *cnf1*, by largely belonging to virotype E (therefore being *papGII*+), and by frequently carrying plasmids with F31 or F36:A4:B1 replicons. This study also describes the presence of *aac(3)-IIa* and of a IS*15DIV*-bounded transposon with *aac(6’)-lb-cr*, *bla*_OXA-1_, Δ*catB3*, although they report the frequent presence of *aac(3)-IId* instead of that of *aac(3)-IIe* (an arrangement similar to that of MB1860TU_A, given that IS*15DIV* is very closely related to IS*26*), and *bla*_CTX-M-15_ with an IS*Ecp1* upstream of and in the same orientation as *bla*_CTX-M-15_, the reversed *wbuC* and a Tn*2* transposon (possibly MB1860TU_B). The concordance between the markers associated with this “C2 subset” and our proposed C2b sublineage, is striking.

We noted the presence of a plasmid in strain A23EC. This plasmid (named pA23EC) was classified as PTU-FE by COPLA and has four replicons, three of which belong to incompatibility group F: F(31,36):A(4,20):B1, which can be annotated as F-:A-:B1. As it happens, PTU-FE F-:A-:B1 is one of four PTU-replicon combinations previously proposed to mediate most of the flow of resistance and virulence genes between food and clinical strains, so the present work supports the idea that the plasmid flow between food and clinical strains is preferentially mediated by a specific subset of plasmids (Balbuena-Alonso et al., 2022), although the presence of A23EC in food may be incidental in this case. The fourth replicon belongs to the Col156 type, which has been previously described in isolates of the C1 subclade, carrying CTX-M-14 or -27 (Kondratyeva et al., 2020) (seen also in **Figure 1**). The presence of Col156 replicons, which are infrequent, in separate subclades of ST131 raises the possibility of plasmid exchanges across ST131 subclades.

Plasmid pA23EC had a complete set of mobilization genes, suggesting that this plasmid is capable of conjugation. Indeed, we detected conjugation at both 20 and 37°C. Conjugation frequencies were similar at both temperatures, which is surprising, as lower temperature slows growth down; however, it appears that the frequency of conjugation of A23EC to the C600^Rif+^ recipient strain is not influenced by incubation temperature. Thus, our report is the first to show efficient conjugation for a subclade C2 strain of ST131 at a temperature under 37°C. These observations suggest that pA23EC has the potential to spread *via* conjugation, not only in human hosts but also in environmental reservoirs. Also note that pA23EC is classified as PTU-FE, which exhibits a host range of III on a six-grade scale, with a level of promiscuity to the level of family, and therefore has the potential to contribute to genetic exchange across multiple species in the Enterobacteriaceae.

A23EC is a multidrug-resistant strain, with resistance to aminoglycosides, penicillin, cephalosporins, tetracyclines, quinolones and monobactam. The prevalence of strains that are resistant to all first-line drugs is rising at an alarming rate (Manges, 2016), and multidrug-resistant ExPEC has been categorized by the WHO as a high-risk pathogen of critical priority (Tacconelli et al., 2018). Genotypically, we found nine ARGs in A23EC. Six of these mapped to Tn*MB1860* in the chromosome, in two separate TUs. The first TU (MB1860TU_A) carried *aac(6’)-lb-cr*, *bla*_OXA-1_, Δ*catB3*, *aac(3)-IIe* and *tmrB*, whereas the second TU (MB1860TU_B) carried *bla*_CTX-M-15_. Two additional ARGs were found elsewhere in the chromosome >1 Mb away from TnMB1860, namely *aac(3)-IIa* and *mdfA*; *tetA/tetR* were the only ARGs found in the pA23EC plasmid.

The ARGs that we identified are likely responsible for the observed resistance to penicillins and first-generation cephalosporins (*bla*_OXA-1_ and *bla*_CTX-M-15_), to synthetic cephalosporins (*bla*_CTX-M-15_), monobactams (*bla*_CTX-M-15_), (Zhu et al., 2022), fluoroquinolones (*aac(6’)-lb-cr*, and mutations at position S83L, D87N of gyrase and position S80L of ParC (Redgrave et al., 2014; Huseby et al., 2017), to gentamicin (*aac(3)-IIe*), to tobramycin (*aac(6’)-lb-cr* and *aac(3)-IIe*), to amikacin (*aac(6’)-lb-cr*, intermediate resistance) (Ojdana et al., 2018; Stogios et al., 2022) and to tetracycline (*tetA*/*tetR*) (Møller et al., 2016).

The genes *mdfA* and *tmrB* could enhance resistance to a variety of drugs rather than being primarily responsible for resistance to a specific drug. *MdfA* (also known as *cmlA* or c*mr*) is a proton-dependent pump with a very wide range of substrates that include chloramphenicol, erythromycin, roxithromycin and certain aminoglycosides and fluoroquinolones (Edgar and Bibi, 1997). TmrB is an ATP-binding membrane protein that protects against tunicamycin exposure, binding tunicamycin and functioning either as an efflux pump or as a permeability barrier for this drug (Noda et al., 1992; Noda et al., 1995). Given that tunicamycin is an experimental drug not used as an antibiotic in the clinic or as growth promoter for animals, the frequent presence of the *tmrB* gene in resistance-determining regions (Grevskott et al., 2020; Pungpian et al., 2022) is intriguing and points to a possible role as modulator of resistance to other antibiotics.

Chloramphenicol acetyl transferases acetylate the antibiotic chloramphenicol at the 30-hydroxyl position using acetyl coenzyme as an acetyl donor (White et al., 1999). CatB3 is a B-type acetyltransferase, which tends to have low activity against chloramphenicol, and forms homotrimers. Trimer formation requires the C-terminal α-helical domain and is important for catalysis because the acetyl acceptor site of each protein is located in a pocket formed between monomers of the trimer. Therefore, the loss of the C-terminal α-helical domain in A23EC’s Δ*catB3* is expected to destabilize the trimer (Alcala et al., 2020). Belonging to a CAT family with low activity against chloramphenicol to begin with and having a truncation that likely suppresses its catalytic activity could explain the sensitivity of A23EC to chloramphenicol despite carrying Δ*catB3*. Indeed, in a previous report, a strain with this exact truncation was reported as sensitive to chloramphenicol (Hubbard et al., 2020). However, the fact that the two deletions observed in Δ*catB3* of A23EC are in-frame, and that this allele is fully conserved in our proposed C2b sublineage and even in other STs (strain CP048934.1 is classified as ST315) suggests that *ΔcatB3* might retain some residual function that is being maintained through selection.

One of the C2b genomes (strain 11A CP049077.2) carries the Tn*MB1860* transposon integrated in the chromosome and also in plasmid p11A_2. This results in a gene dosage duplication for all the genes encoded in Tn*MB1860* that likely makes this transposon structure and all the ARGs that it contains redundant in strain 11A. The concurrent presence of a given ARG in a chromosome and in a plasmid within the same isolate is not uncommon and is interpreted as an intermediate stage in the incorporation of genetic content from a plasmid into the chromosome (Wang et al., 2022). This interpretation is also consistent with the incomplete penetrance of the F(31,36):A(4,20):B1 plasmid in sublineage C2b, which appears to have been lost independently four times (**Figure 1**).

The A23EC strain bears the seven virulence markers that are characteristic of ExPEC, as expected for ST131 (**Figure S1**) (Johnson et al., 2003; van Hoek et al., 2016); the presence of *chuA*, *fyuA*, and *yfcV* identifies A23EC as a potentially UPEC strain and its *papGII+* status suggests it may be particularly virulent. Indeed, the PapGII fimbrial tip adhesin was shown to promote inflammation in renal tissue through transcriptional reprogramming of kidney cells (Biggel et al., 2020), and *papGII+* strains have been reported to be enriched in blood isolates relative to urine/UTI infections (Ambite et al., 2019). Other virulence genes in A23EC including P fimbriae, hemolysins, siderophores, toxins and capsular synthesis, all located within PAIs, have previously been linked to the development of invasive infections (Sabaté et al., 2006; Tsoumtsa Meda et al., 2022). Outside PAIs, we identified *fimH*, which is associated with adherence on biotic and abiotic surfaces (Cookson et al., 2002), and *iss, sitA* and *ompT*, which are genes associated with serum resistance and favoring colonization and invasion mainly described in APEC strains (Olsen et al., 2012), as well as the presence of toxin gene *usp* associated with strains causing pyelonephritis, prostatitis and bacteremia (Nipič et al., 2013).

A high virulence of the C2b sublineage of the C2 subclade is also supported by the sources of isolation of C2b samples. Out of eighteen samples, ten were isolated from patients suffering from septicemia (58.8%) and none from urine. For comparison, out of thirteen C2a samples, five were isolated from urine (38.5%) and only one was isolated from the bloodstream (7.7%). Consistent with these observations, Pajand et al., 2021 report that the “C2 subset” (which as explained above aligns with our proposed C2b sublineage) accounted for the excess resistance and virulence of subclade C2 relative to C1 subclade strains (Pajand et al., 2021). However, the observed difference in sample origin between the C2a and C2b sublineages of the C2 subclade could also be attributed to unknown variables affecting the sampling and is based in both cases on a small number of samples.

Based on epidemiological surveillance studies that have been carried out in different parts of the world in the “One Health” context, non-animal agricultural products have been proposed to be important vectors for the circulation of multidrug-resistant ExPEC strains between humans, animals and the environments (Meena et al., 2023). Our report of an isolate belonging to the C2 subclade of ST131 contaminating green leafy vegetables adds support to this hypothesis. We searched for additional relevant reports in the literature. **Table S4** lists examples of *E. coli* strains isolated from vegetables, along with their phylotypes (when known) and their corresponding reference. A few sequence types stand out, namely ST10, ST38, ST23, ST69 and ST155, which to our knowledge have been independently reported 8, 4, 3, 3, and 3 times, respectively out of a total of 36 annotated examples. Note that while most of the isolates reported likely represent commensals (based on their phylotype), about ⅓ of them (including ST69) belong to likely ExPEC phylotypes (B2, and D), supporting the idea that agricultural products can indeed serve as vectors for the transmission of ExPEC strains.

Humans represent the main reservoirs of *E. coli* ST131 by colonizing the intestine (Morales Barroso et al., 2018; Sarkar et al., 2018; Johnson et al., 2022). When isolated in foods, the source of ST131 *E. coli* are typically animal products such as meat and dairy products (Platell et al., 2011; Liu et al., 2018) and the C2 (O25:H4/H30) subclade of *E. coli* is much less frequent in foods other ST131 lineages such as H22 (Manges, 2016; Massella et al., 2020). The isolation of a *bla*_CXT-M-15_-bearing ST131 strain from an agricultural product has only been previously reported once, from a bitter cucumber imported from the Dominican Republic (Müller et al., 2016), and we ignore further details about its molecular classification. Thus, to our knowledge, this is the first report of an ST131 clone belonging to the C2 subclade (O25:H4/H30) contaminating green leafy vegetable. The isolation of an emerging, potentially uropathogenic strain of ST131 from spinach is relevant to public health because the consumption of fresh produce has increased as a result of a healthier lifestyle (Castro-Rosas et al., 2012).

Three other strains of the C2b sublineage described here were isolated from wastewater (**Figure 1**). Further, ST131 clade C strains have been observed to survive wastewater treatment and release to surface water (Tausova et al., 2012; Zurfluh et al., 2013; Müller et al., 2016). Thus, A23EC could have reached spinach *via* contaminated water. Admittedly, the present study cannot determine the point at which A23EC contaminated the spinach, and it is therefore possible that contamination happened after the spinach was harvested (during processing, transport or at the supermarket itself) but we can say that this strain is able to persist in spinach long enough for transmission. Whether the ability of A23EC and possibly other C2b strains to persist in fresh vegetables represents a new adaptation or it was simply previously missed is unclear. The presence of virulence factors facilitating adherence to human and animal cells (*papABCD*, *papGII*, *iha*, *yfcV*, *kpsm-TII-k5*, *fimABCDEFGHI* and *csgABCDEFG*) (Sarowska et al., 2019) may be relevant. This is particularly true of adhesins CsgA and CsgB, which have been described to be significantly involved in adhesion and colonization in spinach leaves (Saldaña et al., 2011; MacArisin et al., 2012), and allow their proliferation on the food surface through the formation of biofilms (Zhao et al., 2022). In addition, previous studies have demonstrated the ability of *E. coli* strains to reside within the internal cavity of stomata and internal tissues; this internalization protects the bacteria from disinfecting and bactericidal products, thereby increasing their survival. Internalization also contributes to inefficient washing and sanitizing treatments in vegetables (Gullian-Klanian and Sánchez-Solis, 2018; Querido et al., 2020). However, these previous studies have focused on STEC and EHEC strains and may have missed the presence of ExPEC in these foods.

Some reports interpret the occurrence of ST131 strain in different hosts such as companion and food animals or in different environmental niches such as sewage and other aquatic environments as overflow from its main niche (Melo et al., 2019; Finn et al., 2020); in contrast, other studies report specialization in ST131 strains depending on their origin, suggesting that they are adapting to different niches. This raises complex questions about the role of reservoirs in ST131 evolution and spread (Bonnet et al., 2021; Denamur et al., 2021). The isolation of ST131 strains in foods such as A23EC paves the ground for understanding the epidemiology and evolutionary dynamics of ST131 through rigorous and systematic monitoring using selected molecular markers.

In conclusion, our results add to previous knowledge about the global dissemination of ST131 by confirming the emergence of a distinct sublineage of subclade C2 (C2b), that has the potential to be highly pathogenic and that bears a transposon structure that has the potential to facilitate the evolution of carbapenem resistance among non-carbapenemase-producing enterobacterales (Shropshire et al., 2021). The genetic content of virulence and resistance genes and associated mobilization elements described here for the A23EC strain, together with the plasticity of *E. coli* ST131 genome, suggests that this ExPEC strain has the potential to evolve persistence in new environments and to infect humans and/or animals through new routes of transmission. These observations call for a more comprehensive surveillance and monitoring system for ExPEC strains in non-clinical settings.

## Data availability statement

The datasets presented in this study can be found in online repositories. The names of the repository/repositories and accession number(s) can be found in the article/**Supplementary material**.

## Author contributions

MB-A organized the database and performed the formal analysis. MB-A, GC-C, EC-L, and MC participated in the research. MC, PL-Z, and RR-G supervised the experimental and bioinformatics analysis. MC performed extensive review of the original draft. RR-G performed resources and project administration and funding acquisition. All authors contributed to the article and approved the submitted version.
