# Supplementary material for: Strain belonging to an emerging, virulent sublineage of ST131 Escherichia coli isolated in fresh spinach, suggesting that ST131 may be transmissible through agricultural products
Source: Front Cell Infect Microbiol. 2023 Oct 9;13:1237725. doi: 10.3389/fcimb.2023.1237725 (PMC10591226; doi:10.3389/fcimb.2023.1237725)
Supplement: Supplementary file 1 [file DataSheet_1.docx]

Supplementary Material

Strain belonging to an emerging, virulent sublineage of ST131 *Escherichia coli* isolated in fresh spinach, suggesting that ST131 may be transmissible through agricultural products.

Maria G. Balbuena-Alonso, Gerardo Cortés-Cortés, Manel Camps, Eder A. Carreón-León, Patricia Lozano-Zarain, Rosa del Carmen Rocha-Gracia

*** Correspondence:** Rosa del Carmen Rocha Gracia, [rochagra@yahoo.com](mailto:rochagra@yahoo.com), rosa.rocha@correo.buap.mx

# Supplementary Figure


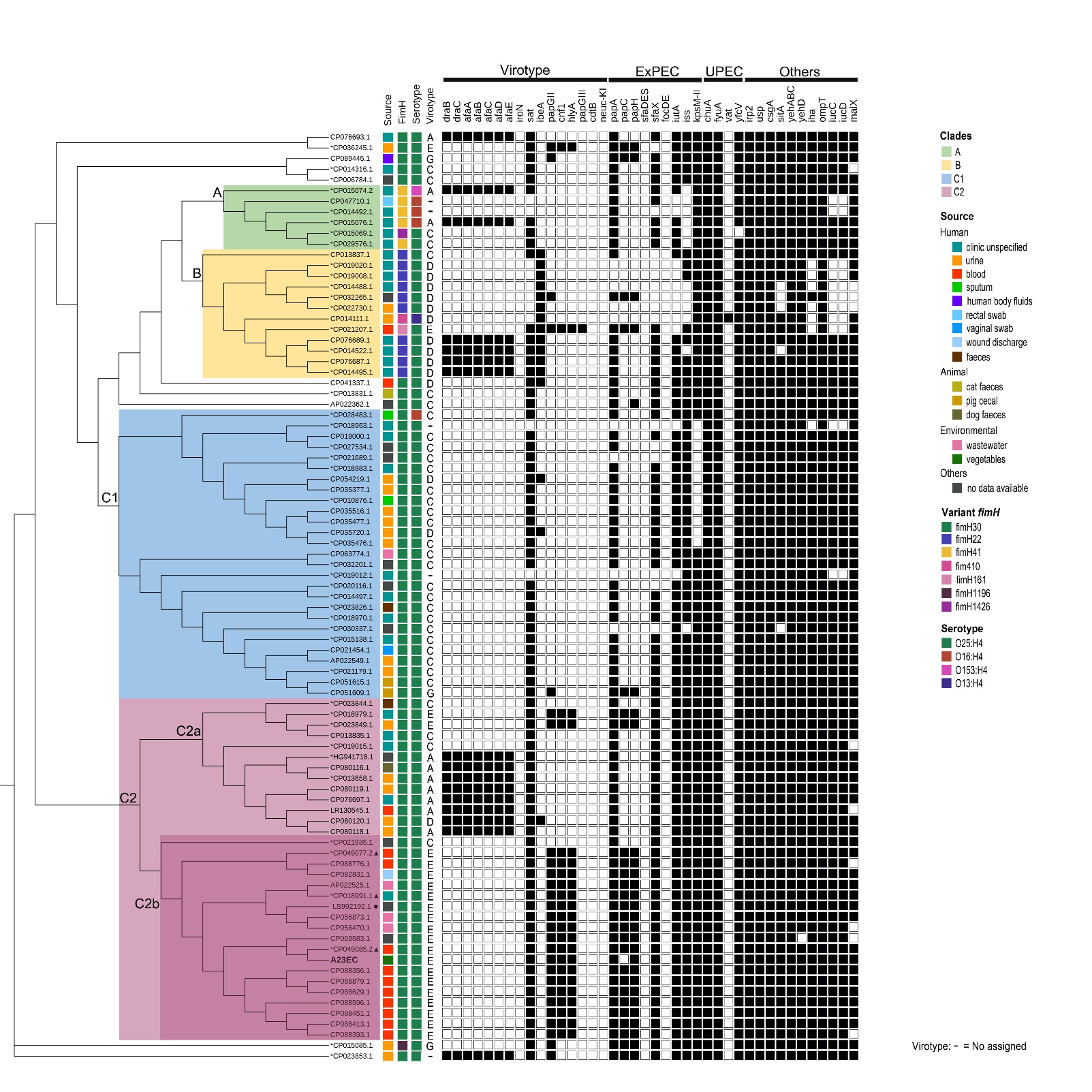


**Figure S1. Correspondence of virulence genes to ST131 lineages.** The phylogeny of the genomes included in this study (86 complete genomes of database GenBank and strain A23EC) is shown in the form of a cladogram. This graphic representation was generated using the CSI Phylogeny platform with EC598 (NZ_HG941718.1) as a reference genome. Filled boxes indicate presence and the incomplete boxes indicate partial presence. The first column indicates the source of isolation. The second column indicates the allelic variant of FimH identified. The third column indicates the serotype determined by Serotypefinder. The fourth column shows the virotype assigned. The 5th to 44th columns indicate a heatmap of virulence genes; columns #5-20 list the markers of virotype; columns #21-29 markers of ExPEC; columns #30-33, markers of UPEC, and columns #34-44 other virulence genes. The asterisk next to the *GenBank* accession number indicates strains contained in the study of Biggel et al., 2022 and those with a small triangle are those belonging to the L1 sublineage. The circle indicates strains included in the study of Shropshire et al., 2021.

**
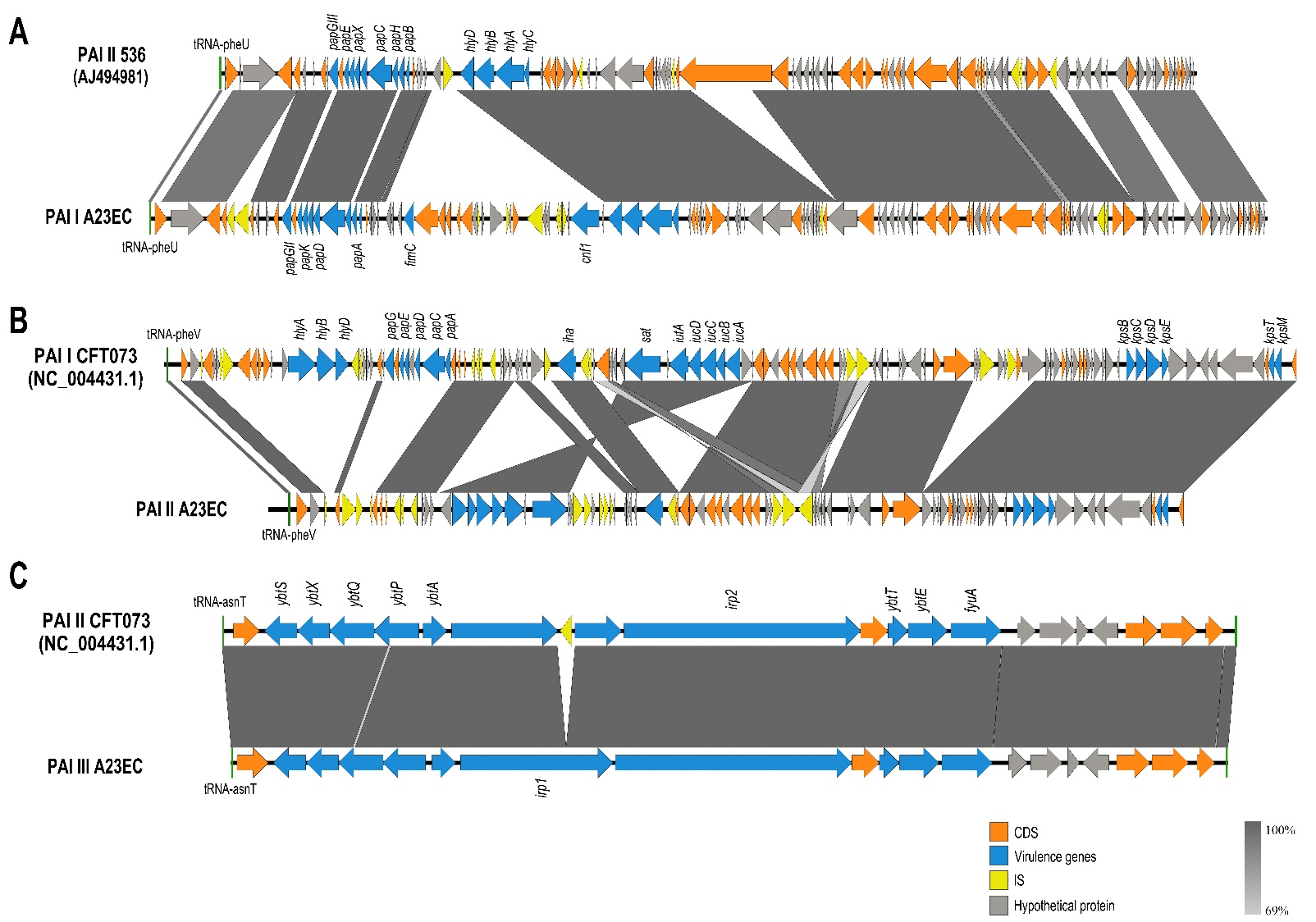
**

**Figure S2. Comparison of the genetic structure of the three pathogenicity islands found in A23EC to the prototype sequence of PAIs CFT073-I, II and 536-II**. **A. PAI I A23EC/ II536 comparison.** With 84% coverage, this comparison shows the presence of thirteen genes on both islands except *cnf1* and FimC present only in A23EC. **B.** **PAI- II A23EC/I CFT073-I comparison**. With 89% coverage, shows the two PAIs sharing thirteen virulence genes, with the region containing the *sat/iutA/iucABCD* genes inverted in the A23EC strain.**C.** **PAI-III A23EC/CFT073-II comparison.** With 98% coverage, shows the *irp1* gene intact. Gray lines indicate regions with 69-100% identity by Blastn. The image was generated with EasyFig (default settings). The accession numbers of the sequences of the prototype strain used were placed in parentheses.

**
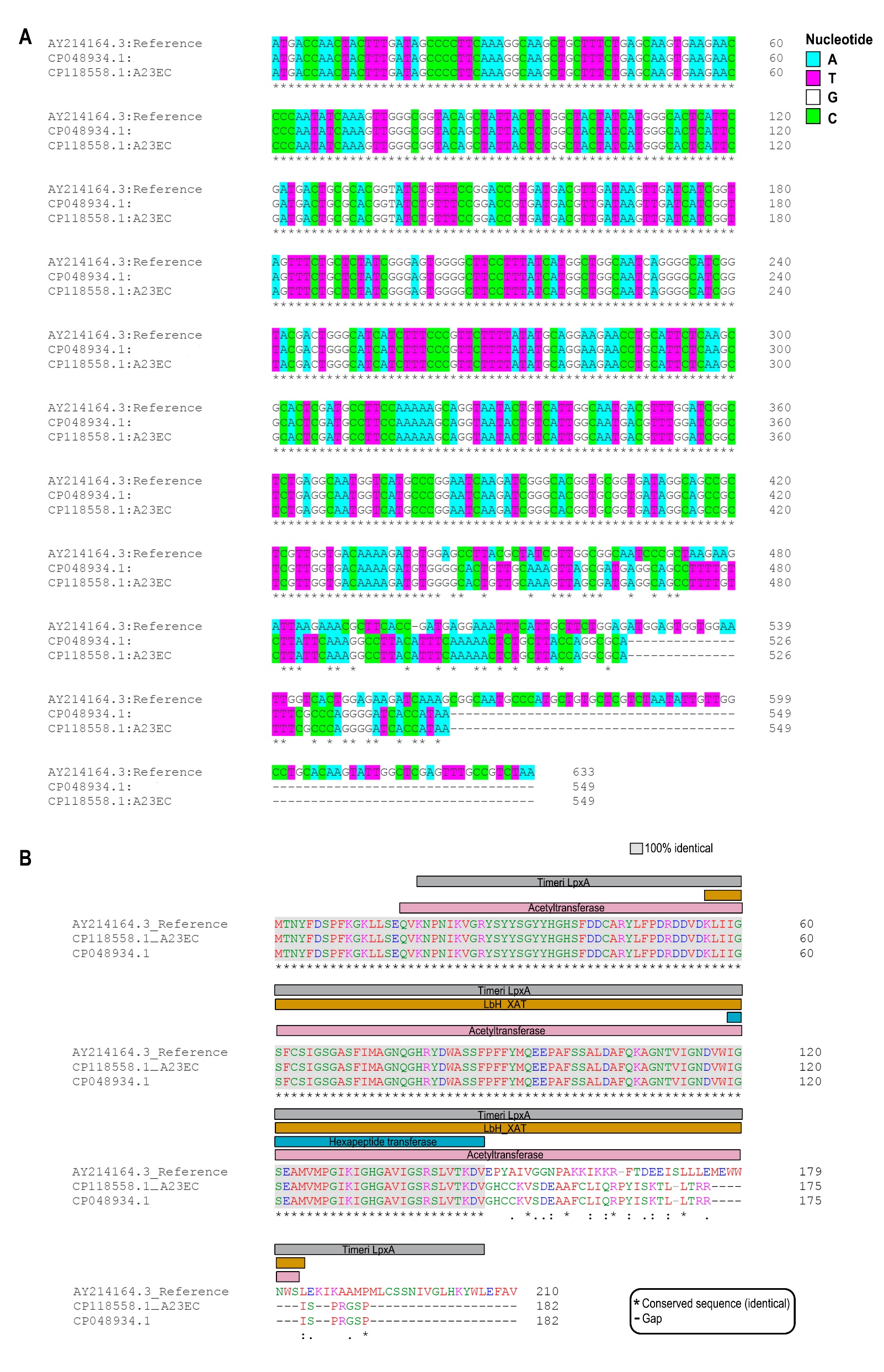
**

**Figure S3. Alignment between the truncated *cat3B* sequence found in A23EC and its reference. A. nucleotide alignment. B. Amino acid alignment.**  The sequence with accession number CP048934.1 is as reported by Hubbard et al., 2020.

**
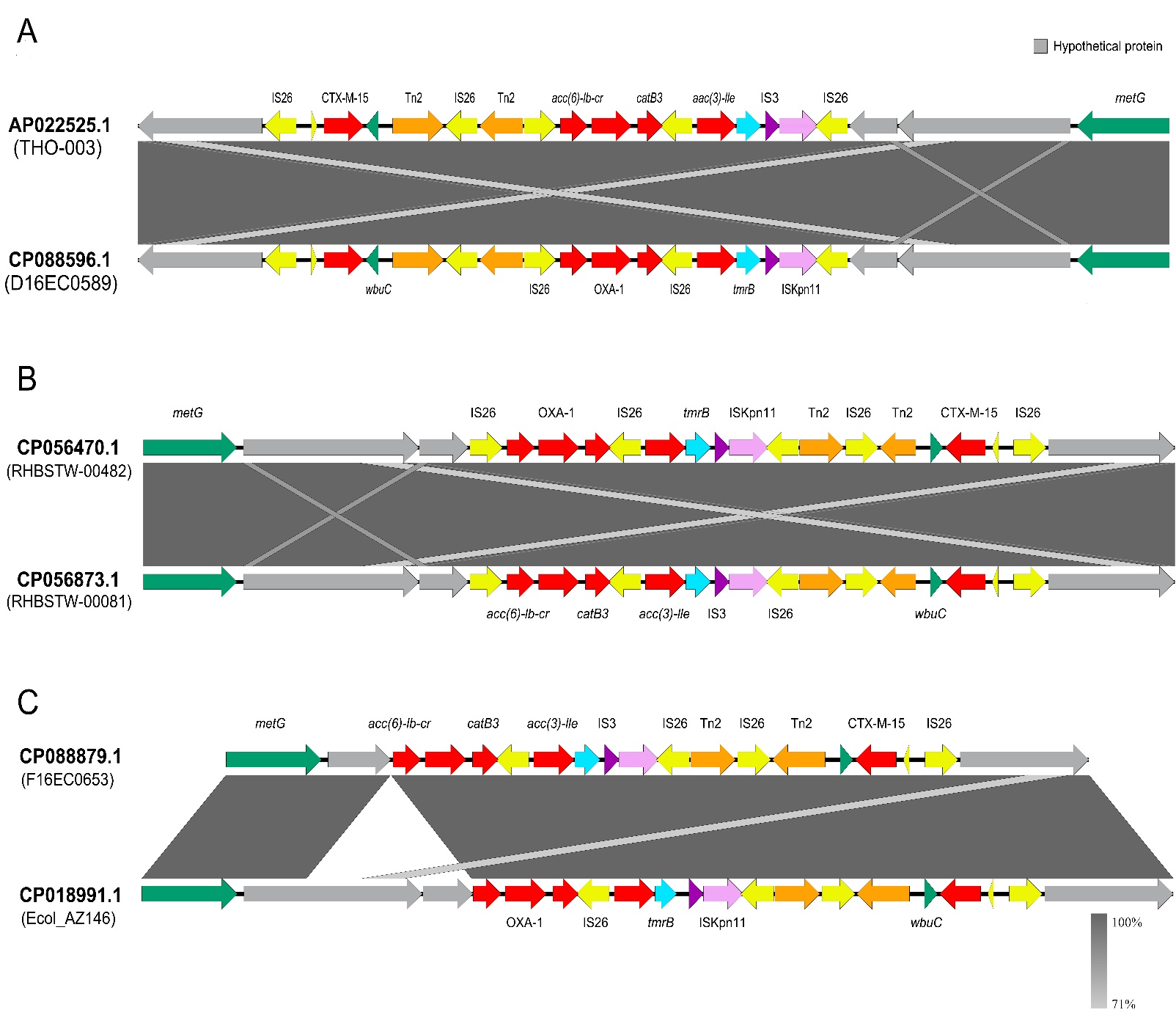
**

**Figure S4. Alignment of genomes contained in clusters 2, 4 and 5. A.** Cluster 2 contains 2 sequences, with MB1860TU_B translocated just upstream of MB1860TU_A in reverse direction, with >99% identity between them. **B**. Cluster 4, with two sequences that maintain >99% between them. **C.** Cluster 5, with two sequences, also exhibits a loss of IS26 at the right end of the TnMB1860TU_A region, in addition, between them in CP018991.1 there is an insertion of 3,509 bp unlike CP088879.1.
