# Supplementary material for: Strain belonging to an emerging, virulent sublineage of ST131 Escherichia coli isolated in fresh spinach, suggesting that ST131 may be transmissible through agricultural products
Source: Front Cell Infect Microbiol. 2023 Oct 9;13:1237725. doi: 10.3389/fcimb.2023.1237725 (PMC10591226; doi:10.3389/fcimb.2023.1237725)
Supplement: Supplementary file 2 [file Table_1.docx]

Supplementary Material

Strain belonging to an emerging, virulent sublineage of ST131 *Escherichia coli* isolated in fresh spinach, suggesting that ST131 may be transmissible through agricultural products.

Maria G. Balbuena-Alonso, Gerardo Cortés-Cortés, Manel Camps, Eder A. Carreón-León, Patricia Lozano-Zarain, Rosa del Carmen Rocha-Gracia

*** Correspondence:** Rosa del Carmen Rocha Gracia, [rochagra@yahoo.com](mailto:rochagra@yahoo.com), rosa.rocha@correo.buap.mx

# Supplementary Table

| **Table S1. 86 complete genomes of *Escherichia coli* ST131 deposited in *GenBank*** | | | | | | | | | | | | |
| --- | --- | --- | --- | --- | --- | --- | --- | --- | --- | --- | --- | --- |
| **Name of Strain** | **Assembly** | **Access number (Chromosome)** | **Clade** | **Bioproject** | **Database** | **Biosample** | **Collection date** | **Location** | **Sample type** | **Source** | **Serotype** | **FimH variant** |
| E302 | GCA_010725305.1 | AP022362.1 | C0 | PRJDB6565 | NCBI Refseq | SAMD00186874 | 2015 | Japan | No data available | No data available | O25:H4 | 30 |
| Ecol_745 | GCA_001618345.2 | CP015074.2 | A | PRJNA316786 | NCBI Refseq | SAMN04590095 | 2012 | Morocco | Clinical | unspecified | O153:H5 | 41 |
| Z247 | GCA_002142715.1 | CP021207.1 | B | PRJNA386074 | NCBI Refseq | SAMN06925129 | 2015 | China | Clinical | blood | O25:H4 | 161 |
| p4A | GCA_011331215.2 | CP049085.2 | C2b | PRJNA603908 | NCBI Refseq | SAMN13948677 | 2015 | USA | Clinical | blood | O25:H4 | 30 |
| S21EC | GCA_018972225.1 | CP076689.1 | B | PRJNA737575 | NCBI Refseq | SAMN19700015 | 2009 | United Kingdom | Clinical | unspecified | O25:H4 | 22 |
| Dog168 | GCA_019428525.1 | CP080116.1 | C2a | PRJNA748602 | NCBI Refseq | SAMN20339880 | 2009 | Kenya | Animal | dog faeces | O25:H4 | 30 |
| M70 | GCA_019428565.1 | CP080118.1 | C2a | PRJNA748602 | NCBI Refseq | SAMN20339878 | 2010 | Czech Republic | Clinical | Urine | O25:H4 | 30 |
| M45 | GCA_019428585.1 | CP080119.1 | C2a | PRJNA748602 | NCBI Refseq | SAMN20339877 | 2010 | Czech Republic | Clinical | Urine | O25:H4 | 30 |
| M24 | GCA_019428605.1 | CP080120.1 | C2a | PRJNA748602 | NCBI Refseq | SAMN20339876 | 2009 | Czech Republic | Clinical | Urine | O25:H4 | 30 |
| BR43-DEC | GCA_004118895.1 | CP035377.1 | C1 | PRJNA511007 | NCBI Refseq | SAMN10748606 | 2015 | Brazil | Clinical | Urine | O25:H4 | 30 |
| SA186 | GCA_003856995.1 | CP022730.1 | B | PRJNA395653 | NCBI Refseq | SAMN07411973 | 2012 | Saudi Arabia | Clinical | Urine | O25:H4 | 22 |
| AR_0089 | GCA_003571825.1 | CP032265.1 | B | PRJNA292904 | NCBI Refseq | SAMN04014930 | No available | No available | No data available | No data available | O25:H4 | 22 |
| Ecol_244 | GCA_002012305.1 | CP019020.1 | B | PRJNA316786 | NCBI Refseq | SAMN05511157 | 2010 | Argentina | Clinical | unspecified | O25:H4 | 22 |
| THO-003 | GCA_015138575.1 | AP022525.1 | C2b | PRJDB9036 | NCBI Refseq | SAMD00196001 | 2018 | Japan | Enviromental | wastewater | O25:H4 | 30 |
| AR_451 | GCA_003288455.1 | CP030337.1 | C1 | PRJNA316321 | NCBI Refseq | SAMN07291544 | No available | No available | No data available | No data available | O25:H4 | 30 |
| RHBSTW-00482 | GCA_013748455.1 | CP056470.1 | C2b | PRJNA605147 | Genbank | SAMN15148629 | 2017 | United Kingdom | Enviromental | wastewater | O25:H4 | 30 |
| RHBSTW-00081 | GCA_013795795.1 | CP056873.1 | C2b | PRJNA605147 | Genbank | SAMN15148474 | 2017 | United Kingdom | Enviromental | wastewater | O25:H4 | 30 |
| O25b:H4-ST131 | GCA_015277555.1 | CP063774.1 | C1 | PRJNA670813 | NCBI Refseq | SAMN16520835 | 2018 | Hong Kong | Enviromental | wastewater | O25:H4 | 30 |
| Ecol_AZ146 | GCA_002012085.1 | CP018991.1 | C2b | PRJNA316786 | NCBI Refseq | SAMN05511152 | 2012 | Italy | Clinical | unspecified | O25:H4 | 30 |
| pA11 | GCA_011330935.2 | CP049077.2 | C2b | PRJNA603908 | NCBI Refseq | SAMN13948684 | 2015 | USA | Clinical | blood | O25:H4 | 30 |
| FDAARGOS_1265 | GCA_016890045.1 | CP069583.1 | C2b | PRJNA231221 | NCBI Refseq | SAMN16357407 | No available | USA | No data available | No data available | O25:H4 | 30 |
| SCAID WND1-2021 | GCA_019915525.1 | CP082831.1 | C2b | PRJNA754843 | NCBI Refseq | SAMN20982447 | 2021 | Kazakhstan | Clinical | wound discharge | O25:H4 | 30 |
| F17EC0098 | GCA_021130495.1 | CP088356.1 | C2b | PRJNA782071 | Genbank | SAMN23391703 | 2017 | South Korea | Clinical | blood | O25:H4 | 30 |
| F16EC0507 | GCA_021130695.1 | CP088393.1 | C2b | PRJNA782071 | Genbank | SAMN23391692 | 2016 | South Korea | Clinical | blood | O25:H4 | 30 |
| F16EC0342 | GCA_021130865.1 | CP088413.1 | C2b | PRJNA782071 | Genbank | SAMN23391686 | 2016 | South Korea | Clinical | blood | O25:H4 | 30 |
| F16EC0121 | GCA_021131025.1 | CP088451.1 | C2b | PRJNA782071 | Genbank | SAMN23391678 | 2016 | South Korea | Clinical | blood | O25:H4 | 30 |
| C17EC0083 | GCA_021132115.1 | CP088629.1 | C2b | PRJNA782071 | Genbank | SAMN23391614 | 2017 | South Korea | Clinical | blood | O25:H4 | 30 |
| B16EC0725 | GCA_021132975.1 | CP088776.1 | C2b | PRJNA782071 | Genbank | SAMN23391567 | 2016 | South Korea | Clinical | blood | O25:H4 | 30 |
| F16EC0653 | GCA_021365955.1 | CP088879.1 | C2b | PRJNA782071 | Genbank | SAMN23391700 | 2016 | South Korea | Clinical | blood | O25:H4 | 30 |
| TO217 | GCA_900520365.1 | LS992192.1 | C2b | PRJEB27475 | NCBI Refseq | SAMEA4830526 | 2018 | No available | No data available | No data available | O25:H4 | 30 |
| Ecol_867 | GCA_002012205.1 | CP018983.1 | C1 | PRJNA316786 | NCBI Refseq | SAMN05511148 | 2013 | Canada | Clinical | unspecified | O25:H4 | 30 |
| Ecol_AZ153 | GCA_002012245.1 | CP019000.1 | C1 | PRJNA316786 | NCBI Refseq | SAMN05511160 | 2013 | China | Clinical | unspecified | O25:H4 | 30 |
| 4/1/2001 | GCA_003856695.1 | CP023844.1 | C2a | PRJNA413669 | NCBI Refseq | SAMN07760931 | 2009 | Sweden | Clinical | feces | O25:H4 | 30 |
| CCUG 73778 | GCA_009577985.1 | CP041337.1 | C0 | PRJNA305687 | NCBI Refseq | SAMN12169096 | 2008 | Sweden | Clinical | blood | O25:H4 | 30 |
| THO-015 | GCA_015139735.1 | AP022549.1 | C1 | PRJDB9036 | NCBI Refseq | SAMD00196013 | 2018 | Japan | Clinical | Urine | O25:H4 | 30 |
| 2019_APHA | GCA_014217135.1 | CP051609.1 | C1 | PRJNA625187 | NCBI Refseq | SAMN14594835 | 2019 | United Kingdom | Animal | Pig,cecal sample | O25:H4 | 30 |
| 2017_APHA | GCA_014217155.1 | CP051615.1 | C1 | PRJNA625187 | NCBI Refseq | SAMN14594834 | 2017 | United Kingdom | Animal | Pig,cecal sample | O25:H4 | 30 |
| EcPF18 | GCA_013372325.1 | CP054219.1 | C1 | PRJNA636382 | NCBI Refseq | SAMN15075997 | 2018 | USA | Clinical | Urine | O25:H4 | 30 |
| Ecol_448 | GCA_001618365.1 | CP015076.1 | A | PRJNA316786 | NCBI Refseq | SAMN04590097 | 2011 | Argentina | Clinical | unspecified | O16:H5 | 41 |
| O25b:H4 | GCA_001874485.1 | CP015085.1 | C2 | PRJNA316859 | NCBI Refseq | SAMN04605558 | 2014 | Saudi Arabia | Clinical | Urine | O25:H4 | 1196 |
| MNCRE44 | GCA_000931565.1 | CP010876.1 | C1 | PRJNA272863 | NCBI Refseq | SAMN03287565 | 2012 | USA | Clinical | sputum | O25:H4 | 30 |
| ZH193 | GCA_001566675.1 | CP014497.1 | C1 | PRJNA307507 | NCBI Refseq | SAMN04381846 | 2004 | No available | Clinical | unspecified | O25:H4 | 30 |
| Ecol_743 | GCA_001618325.1 | CP015069.1 | A | PRJNA316786 | NCBI Refseq | SAMN04590096 | 2012 | United Arab Emirates | Clinical | unspecified | O25:H4 | 1426 |
| Ecol_732 | GCA_001617565.1 | CP015138.1 | C1 | PRJNA316786 | NCBI Refseq | SAMN04621897 | 2012 | Thailand | Clinical | unspecified | O25:H4 | 30 |
| Ecol_276 | GCA_002011965.1 | CP018953.1 | C1 | PRJNA316786 | NCBI Refseq | SAMN05511184 | 2010 | USA | Clinical | unspecified | O25:H4 | 30 |
| Ecol_542 | GCA_002012025.1 | CP018970.1 | C1 | PRJNA316786 | NCBI Refseq | SAMN05511186 | 2011 | Viet Nam | Clinical | unspecified | O25:H4 | 30 |
| Ecol_AZ161 | GCA_002012265.1 | CP019012.1 | C1 | PRJNA316786 | NCBI Refseq | SAMN05511166 | 2013 | USA | Clinical | unspecified | O25:H4 | 30 |
| 81009 | GCA_002157245.1 | CP021179.1 | C1 | PRJNA383781 | NCBI Refseq | SAMN06808253 | 2009 | United Arab Emirates | Clinical | Urine | O25:H4 | 30 |
| 4_4 | GCA_003856655.1 | CP023826.1 | C1 | PRJNA413669 | NCBI Refseq | SAMN07760938 | 2009 | Sweden | Clinical | feces | O25:H4 | 30 |
| E41-1 | GCA_003194345.1 | CP028483.1 | C1 | PRJNA431043 | NCBI Refseq | SAMN08382661 | 2017 | China | Clinical | sputum | O16:H5 | 30 |
| U12A | GCA_004141955.1 | CP035476.1 | C1 | PRJNA516746 | NCBI Refseq | SAMN10817475 | 2012 | USA | Clinical | Urine | O25:H4 | 30 |
| U13A | GCA_004135165.1 | CP035477.1 | C1 | PRJNA516747 | NCBI Refseq | SAMN10817700 | 2013 | USA | Clinical | Urine | O25:H4 | 30 |
| U14A | GCA_004135915.1 | CP035516.1 | C1 | PRJNA516748 | NCBI Refseq | SAMN10836813 | 2015 | USA | Clinical | Urine | O25:H4 | 30 |
| S65EC | GCA_004299805.1 | CP036245.1 | C2 | PRJNA517996 | NCBI Refseq | SAMN10879429 | 2009 | Australia | Clinical | Urine | O25:H4 | 30 |
| S103EC | GCA_018972265.1 | CP076693.1 | C0 | PRJNA737575 | NCBI Refseq | SAMN19700014 | 2010 | Australia | Clinical | unspecified | O25:H4 | 30 |
| MVAST0167 | GCA_001566655.1 | CP014492.1 | A | PRJNA307507 | Genbank | SAMN04381852 | 2010 | USA | Clinical | unspecified | O16:H5 | 41 |
| JJ1886 | GCA_000493755.1 | CP006784.1 | C2 | PRJNA218163 | NCBI Refseq | SAMN02603887 | No available | No available | No data available | No data available | O25:H4 | 30 |
| JJ2434 | GCA_001513635.1 | CP013835.1 | C2a | PRJNA307507 | NCBI Refseq | SAMN04381854 | 2008 | USA | Clinical | unspecified | O25:H4 | 30 |
| Ecol_656 | GCA_002012065.1 | CP018979.1 | C2a | PRJNA316786 | NCBI Refseq | SAMN05511161 | 2012 | USA | Clinical | unspecified | O25:H4 | 30 |
| Ecol_AZ162 | GCA_002012165.1 | CP019015.1 | C2a | PRJNA316786 | NCBI Refseq | SAMN05511185 | 2013 | USA | Clinical | unspecified | O25:H4 | 30 |
| AR_0104 | GCA_002056635.1 | CP020116.1 | C1 | PRJNA292904 | NCBI Refseq | SAMN04014945 | No available | No available | No data available | No data available | O25:H4 | 30 |
| AR_0055 | GCA_002202175.1 | CP021935.1 | C2 | PRJNA292904 | NCBI Refseq | SAMN04014896 | No available | No available | No data available | No data available | O25:H4 | 30 |
| Apr-00 | GCA_003856615.1 | CP023849.1 | C2a | PRJNA413669 | NCBI Refseq | SAMN07760930 | 2009 | Sweden | Clinical | Urine | O25:H4 | 30 |
| AR_0081 | GCA_002996945.1 | CP027534.1 | C1 | PRJNA292904 | NCBI Refseq | SAMN04014922 | No available | No available | No data available | No data available | O25:H4 | 30 |
| U15A | GCA_004193755.1 | CP035720.1 | C1 | PRJNA516749 | NCBI Refseq | SAMN10837069 | 2015 | USA | Clinical | Urine | O25:H4 | 30 |
| S10EC | GCA_018972245.1 | CP076697.1 | C2a | PRJNA737575 | NCBI Refseq | SAMN19700013 | 2009 | United Kingdom | Clinical | unspecified | O25:H4 | 30 |
| EC958 | GCA_000285655.3 | HG941718.1 | C2a | PRJEA61443 | NCBI Refseq | SAMEA2272019 | No available | No available | No data available | No data available | O25:H4 | 30 |
| B36 | GCA_900622635.1 | LR130545.1 | C2a | PRJEB29930 | NCBI Refseq | SAMEA5128441 | No available | Australia | Clinical | blood | O25:H4 | 30 |
| JJ1887 | GCA_001593565.1 | CP014316.1 | C0 | PRJNA311313 | NCBI Refseq | SAMN04481707 | 2007 | USA | Clinical | unspecified | O25:H4 | 30 |
| CD306 | GCA_001513615.1 | CP013831.1 | C0 | PRJNA307507 | NCBI Refseq | SAMN04381845 | 2002 | USA | Animal | cat faeces | O25:H4 | 30 |
| G749 | GCA_001566635.1 | CP014488.1 | B | PRJNA307507 | NCBI Refseq | SAMN04381851 | 2010 | USA | Clinical | unspecified | O25:H4 | 22 |
| H105 | GCA_002193095.1 | CP021454.1 | C1 | PRJNA387731 | NCBI Refseq | SAMN07162922 | No available | Germany | Clinical | veginal swab | O25:H4 | 30 |
| AR_0058 | GCA_002180195.1 | CP021689.1 | C1 | PRJNA292904 | NCBI Refseq | SAMN04014899 | No available | No available | No data available | No data available | O25:H4 | 30 |
| AR_0086 | GCA_003571665.1 | CP032201.1 | C1 | PRJNA292904 | NCBI Refseq | SAMN04014927 | No available | No available | No data available | No data available | O25:H4 | 30 |
| JJ1897 | GCA_001513655.1 | CP013837.1 | B | PRJNA307507 | NCBI Refseq | SAMN04381850 | 2004 | USA | Clinical | unspecified | O25:H4 | 22 |
| Ecol_AZ159 | GCA_002012145.1 | CP019008.1 | B | PRJNA316786 | NCBI Refseq | SAMN05511159 | 2013 | Colombia | Clinical | unspecified | O25:H4 | 22 |
| 5_1 | GCA_021228975.1 | CP089445.1 | C0 | PRJNA787313 | NCBI Refseq | SAMN23798050 | 2021 | China | Clinical | Human body fluids | O25:H4 | 30 |
| uk_P46212 | GCA_001469815.1 | CP013658.1 | C2a | PRJNA297860 | NCBI Refseq | SAMN04159541 | 2005 | United Kingdom | Clinical | Urine | O25:H4 | 30 |
| FDAARGOS_144 | GCA_002944935.1 | CP014111.1 | B | PRJNA231221 | NCBI Refseq | SAMN03996288 | 2014 | USA | Clinical | Urine | O13:H4 | 410 |
| SaT040 | GCA_001566615.1 | CP014495.1 | B | PRJNA307507 | NCBI Refseq | SAMN04381849 | 2007 | USA | Clinical | unspecified | O25:H4 | 22 |
| ZH063 | GCA_001577325.1 | CP014522.1 | B | PRJNA307507 | NCBI Refseq | SAMN04381853 | 2002 | Canada | Clinical | unspecified | O25:H4 | 22 |
| DA33135 | GCA_003181035.1 | CP029576.1 | A | PRJNA472712 | NCBI Refseq | SAMN09245744 | No available | Sweden | Clinical | unspecified | O25:H4 | 30 |
| S22EC | GCA_018972205.1 | CP076687.1 | B | PRJNA737575 | NCBI Refseq | SAMN19700016 | 2009 | United Kingdom | Clinical | unspecified | O25:H4 | 22 |
| D16EC0589 | GCA_021131895.1 | CP088596.1 | C2b | PRJNA782071 | Genbank | SAMN23391627 | 2016 | South Korea | Clinical | blood | O25:H4 | 30 |
| Eco-15 | GCA_015910185.1 | CP047710.1 | A | PRJNA599404 | NCBI Refseq | SAMN13747522 | 2015 | Canada | Clinical | rectal | O16:H5 | 30 |
| 2_0 | GCA_003856635.1 | CP023853.1 | C0 | PRJNA413669 | NCBI Refseq | SAMN07760925 | 2009 | Sweden | Clinical | Urine | O25:H4 | 30 |
