# Supplementary material for: Strain belonging to an emerging, virulent sublineage of ST131 Escherichia coli isolated in fresh spinach, suggesting that ST131 may be transmissible through agricultural products
Source: Front Cell Infect Microbiol. 2023 Oct 9;13:1237725. doi: 10.3389/fcimb.2023.1237725 (PMC10591226; doi:10.3389/fcimb.2023.1237725)
Supplement: Supplementary file 3 [file Table_2.docx]

Supplementary Material

Strain belonging to an emerging, virulent sublineage of ST131 *Escherichia coli* isolated in fresh spinach, suggesting that ST131 may be transmissible through agricultural products.

Maria G. Balbuena-Alonso, Gerardo Cortés-Cortés, Manel Camps, Eder A. Carreón-León, Patricia Lozano-Zarain, Rosa del Carmen Rocha-Gracia

*** Correspondence:** Rosa del Carmen Rocha Gracia, [rochagra@yahoo.com](mailto:rochagra@yahoo.com), rosa.rocha@correo.buap.mx

# Supplementary Tables

| **Table S2. *E. coli* genomes positive for the presence of the region TnMB1860** | | | | | | | | | | |
| --- | --- | --- | --- | --- | --- | --- | --- | --- | --- | --- |
| **Strain** | **Assembly** | **Access number (Chromosome)** | **Bioproject** | **Database** | **Sample** | **Colletion date** | **Location** | **Cluster (figure 3)** | **Plasmid (F-:A-B1) name** | **Plasmid (F-:A-B1) access number** |
| THO-003 | GCA_015138575.1 | AP022525.1 | PRJDB9036 | NCBI RefSeq | wastewater | 2018 | Chromosome | 2 | Contains no plasmid subtype F-:A-:B1 | Contains no plasmid subtype F-:A-:B1 |
| Ecol_AZ146 | GCA_002012085.1 | CP018991.1 | PRJNA316786 | NCBI RefSeq | Clinical sample | 2012 | Chromosome | 5 | pECAZ146_1 | CP018990.1 |
| p11A | GCA_011330935.2 | CP049077.2 | PRJNA603908 | NCBI RefSeq | Bacteremia | 2015 | Chromosome | 3 | p11A_p2 | CP049079.2 |
| p11A_p2 | GCA_011330935.2 | CP049079.2 | PRJNA603908 | NCBI RefSeq | Bacteremia | 2015 | Plasmid | 1 | NA | NA |
| p4A | GCA_011331215.2 | CP049085.2 | PRJNA603908 | NCBI RefSeq | Bacteremia | 2015 | Chromosome | 3 | Contains no plasmid subtype F-:A-:B1 | Contains no plasmid subtype F-:A-:B1 |
| RHBSTW-00482 | GCA_013748455.1 | CP056470.1 | PRJNA605147 | Genbank | Freshwater sample from downstream of wastewater treatment plant | 2017 | Chromosome | 4 | Contains no plasmid subtype F-:A-:B1 | Contains no plasmid subtype F-:A-:B1 |
| RHBSTW-00081 | GCA_013795795.1 | CP056873.1 | PRJNA605147 | Genbank | wastewater effluent | 2017 | Chromosome | 4 | Contains no plasmid subtype F-:A-:B1 | Contains no plasmid subtype F-:A-:B1 |
| SCAID WND1-2021 | GCA_019915525.1 | CP082831.1 | PRJNA754843 | NCBI Refseq | Wound discharge | 2021 | Chromosome | 3 | pSCAID_1 | CP082832.1 |
| F17EC0098 | GCA_021130495.1 | CP088356.1 | PRJNA782071 | NCBI Refseq | Bloodstream infection | 2017 | Chromosome | 3 | pF17EC0098-1 | CP088357.1 |
| F16EC0507 | GCA_021130695.1 | CP088393.1 | PRJNA782071 | NCBI Refseq | Bloodstream infection | 2016 | Chromosome | 3 | pF16EC0507-1 | CP088394.1 |
| F16EC0342 | GCA_021130865.1 | CP088413.1 | PRJNA782071 | NCBI Refseq | Bloodstream infection | 2016 | Chromosome | 3 | pF16EC0342-1 | CP088411.1 |
| F16EC0121 | GCA_021131025.1 | CP088451.1 | PRJNA782071 | NCBI Refseq | Bloodstream infection | 2016 | Chromosome | 3 | pF16EC0121-1 | CP088450.1 |
| D16EC0589 | GCA_021131895.1 | CP088596.1 | PRJNA782071 | NCBI Refseq | Bloodstream infection | 2016 | Chromosome | 2 | Contains no plasmid subtype F-:A-:B1 | Contains no plasmid subtype F-:A-:B1 |
| C17EC0083 | GCA_021132115.1 | CP088629.1 | PRJNA782071 | NCBI Refseq | Bloodstream infection | 2017 | Chromosome | 3 | pC17EC0083-1 | CP088630.1 |
| FDAARGOS_1265 | GCA_016890045.1 | CP069583.1 | PRJNA231221 | NCBI Refseq | No data available | No available | Chromosome | 3 | pFDAARGOS_1265_1 | CP069584.1 |
| B16EC0725 | GCA_021132975.1 | CP088776.1 | PRJNA782071 | NCBI Refseq | Bloodstream infection | 2016 | Chromosome | 3 | pB16EC0725-2 | CP088778.1 |
| F16EC0653 | GCA_021365955.1 | CP088879.1 | PRJNA782071 | Genbank | Bloodstream infection | 2016 | Chromosome | 5 | pF16EC0653-2 | CP088881.1 |
| TO217 | GCA_900520365.1 | LS992192.1 | PRJEB27475 | NCBI Refseq | No data available | 2018 | Chromosome | 3 | pTO217_2 | LS992193.1 |
