# Supplementary material for: Strain belonging to an emerging, virulent sublineage of ST131 Escherichia coli isolated in fresh spinach, suggesting that ST131 may be transmissible through agricultural products
Source: Front Cell Infect Microbiol. 2023 Oct 9;13:1237725. doi: 10.3389/fcimb.2023.1237725 (PMC10591226; doi:10.3389/fcimb.2023.1237725)
Supplement: Supplementary file 4 [file Table_3.docx]

Supplementary Material

Strain belonging to an emerging, virulent sublineage of ST131 *Escherichia coli* isolated in fresh spinach, suggesting that ST131 may be transmissible through agricultural products.

Maria G. Balbuena-Alonso, Gerardo Cortés-Cortés, Manel Camps, Eder A. Carreón-León, Patricia Lozano-Zarain, Rosa del Carmen Rocha-Gracia

*** Correspondence:** Rosa del Carmen Rocha Gracia, [rochagra@yahoo.com](mailto:rochagra@yahoo.com), rosa.rocha@correo.buap.mx

# Supplementary Tables

**Table S3. Antimicrobial resistance phenotypic profile for *E. coli* strain A23EC.**

| **Antibiotic (Class)^a^** | **Abbreviations** | **Disc load (µg)** | **Interpretative standard^b^** | | |
| --- | --- | --- | --- | --- | --- |
|  |  |  | **S** | **I** | **R** |
| Amikacin (A) | AN | 30 |  |  |  |
| Gentamycin (A) | GM | 10 |  |  |  |
| Streptomycin (A) | S | 10 |  |  |  |
| Tobramycin (A) | NN | 10 |  |  |  |
| Ampicillin (P) | AM | 10 |  |  |  |
| Amoxicillin / clavulanic acid (P) | AMC | 20/10 |  |  |  |
| Cefuroxime (C) | CXM | 30 |  |  |  |
| Cefotaxime (C) | CTX | 30 |  |  |  |
| Ceftazidime (C) | CAZ | 30 |  |  |  |
| Cefepime (C) | FEP | 30 |  |  |  |
| Cefoxitin (C) | FOX | 30 |  |  |  |
| Aztreonam (M) | ATM | 30 |  |  |  |
| Trimethoprim (FI) | TMP | 5 |  |  |  |
| Trimethoprim / sulfamethoxazole (FI) | SXT | 1.25/23.75 |  |  |  |
| Nalidixic acid (Q) | NA | 30 |  |  |  |
| Ciprofloxacin (F) | CIP | 5 |  |  |  |
| Chloramphenicol (Ph) | C | 30 |  |  |  |
| Tetracycline (T) | TET | 30 |  |  |  |
| Meropenem (Ca) | MEM | 10 |  |  |  |
| Imipenem (Ca) | IMP | 10 |  |  |  |
| Fosfomycin (Pho) /G6P | FOS | 200/50 |  |  |  |

^a^Class A, Amynoglicosides; B, betalactams; C, Cephalosporin; Ca, Carbapenems; F, Fluoroquinolones; FI, Folate inhibitors; M, Monobactam; P, Penicillins; Ph, Phenicols; Q, Quinolones; T, Tetracyclin; Pho, Phosphonic.
