# Supplementary material for: Strain belonging to an emerging, virulent sublineage of ST131 Escherichia coli isolated in fresh spinach, suggesting that ST131 may be transmissible through agricultural products
Source: Front Cell Infect Microbiol. 2023 Oct 9;13:1237725. doi: 10.3389/fcimb.2023.1237725 (PMC10591226; doi:10.3389/fcimb.2023.1237725)
Supplement: Supplementary file 5 [file Table_4.docx]

Supplementary Material

Strain belonging to an emerging, virulent sublineage of ST131 *Escherichia coli* isolated in fresh spinach, suggesting that ST131 may be transmissible through agricultural products.

Maria G. Balbuena-Alonso, Gerardo Cortés-Cortés, Manel Camps, Eder A. Carreón-León, Patricia Lozano-Zarain, Rosa del Carmen Rocha-Gracia

*** Correspondence:** Rosa del Carmen Rocha Gracia, [rochagra@yahoo.com](mailto:rochagra@yahoo.com), rosa.rocha@correo.buap.mx

# Supplementary Tables

**Table S4. Reports of ExPEC strains in vegetables.**

| **Source** | **ST** | **Phylogroup** | **Location** | **Year** | **Reference** |
| --- | --- | --- | --- | --- | --- |
| Barley,Oat, Fennel and Parsley | ST10 | A | Tunisia | 2012-2013 | (Ben Said et al., 2015) |
|  | ST23 | A |  |  |  |
|  | ST58 | B1 |  |  |  |
|  | ST117 | D |  |  |  |
| Bitter cucumber, basil, beans, chilli, coriander, curry leaves and okra | ST10 | A | India, Thailand, Vietnam, and the Dominican Republic | 2014 | (Zurfluh et al., 2015) |
|  | ST23 | A |  |  |  |
|  | ST38 | D |  |  |  |
|  | ST58 | B1 |  |  |  |
|  | ST155 | B1 |  |  |  |
|  | ST405 | D |  |  |  |
| Basil, beans, bitter cucumber cha-om, coriander, chili, curry leaves and okra | ST10 | A | India, Thailand, Vietnam, and the Dominican Republic | 2014 | (Müller et al., 2016) |
|  | ST23 | A |  |  |  |
|  | ST38 | D |  |  |  |
|  | ST131 | B2 |  |  |  |
|  | ST155 | B1 |  |  |  |
|  | ST405 | D |  |  |  |
| Lettuce | ST10 | A | Portugal | 2014 | (Araújo et al., 2017) |
|  | ST48 | A |  |  |  |
|  | ST155 | B1 |  |  |  |
|  | ST424 | B1 |  |  |  |
|  | ST446 | B1 |  |  |  |
|  | ST2522 | B1 |  |  |  |
| Kale and lettuce | ND | ND | USA | 2015 | (Scheinberg et al., 2017) |
| Lettuce and tomato | ST48 | A | China | 2015-2016 | (Luo et al., 2017) |
|  | ST69 | D |  |  |  |
| Tomato, radish, mung sprout and zucchini | ND | ND | Czech Republic | 2015 | (Janalíková et al., 2018) |
| Parsley and alfalfa | ST410 | C | Ecuador | 2015 | (Ortega-Paredes et al., 2018) |
| Lettuce | ND | ND | Northern California, USA | 2018-2019 | (Liao et al., 2020) |
| Vegetables leafy | ST10 | A | Japan | 2018 | (Song et al., 2020) |
|  | ST38 | D |  |  |  |
|  | ST69 | D |  |  |  |
|  | ST354 | F |  |  |  |
| *Aster scaber*, Mapleleaf, water parsley | ST10 | A | South Korea | 2018 | (Chelaghma et al., 2021) |
|  | ST38 | D |  |  |  |
|  | ST69 | D |  |  |  |
| Lettuce | ST10 | A | South Korea | 2018 | (Oh et al., 2020) |
| Celery, parsley, and salad roman | ST10 | A | Bolinia, Italy | 2017-2018 | (Massella et al., 2021) |
|  | ST88 | C |  |  |  |
|  | ST3601 | B2 |  |  |  |
| Cucumber, carrot, tomate, radish,chilli,fenugreek,coriander,peppermint,spring onoin, cabagge and spinach | ND | ND | Rajasthan, India | 2018-2022 | (Priyanka et al., 2023) |
