# Supplementary material for: Strain belonging to an emerging, virulent sublineage of ST131 Escherichia coli isolated in fresh spinach, suggesting that ST131 may be transmissible through agricultural products
Source: Front Cell Infect Microbiol. 2023 Oct 9;13:1237725. doi: 10.3389/fcimb.2023.1237725 (PMC10591226; doi:10.3389/fcimb.2023.1237725)
Supplement: Supplementary file 6 [file Table_5.docx]

Supplementary Material

References of supplementary material (Table S4)

Strain belonging to an emerging, virulent sublineage of ST131 *Escherichia coli* isolated in fresh spinach, suggesting that ST131 may be transmissible through agricultural products.

Maria G. Balbuena-Alonso, Gerardo Cortés-Cortés, Manel Camps, Eder A. Carreón-León, Patricia Lozano-Zarain, Rosa del Carmen Rocha-Gracia

*** Correspondence:** Rosa del Carmen Rocha Gracia, [rochagra@yahoo.com](mailto:rochagra@yahoo.com), [rosa.rocha@correo.buap.mx](mailto:rosa.rocha@correo.buap.mx)

Ben Said L, Jouini A, Klibi N, Dziri R, Alonso CA, Boudabous A, Ben Slama K, Torres C. Detection of extended-spectrum beta-lactamase (ESBL)-producing Enterobacteriaceae in vegetables, soil and water of the farm environment in Tunisia. *Int J Food Microbiol*. 2015 Jun 16;203:86-92. doi: 10.1016/j.ijfoodmicro.2015.02.023.

Zurfluh K, Nüesch-Inderbinen M, Morach M, Zihler Berner A, Hächler H, Stephan R. Extended-spectrum-β-lactamase-producing Enterobacteriaceae isolated from vegetables imported from the Dominican Republic, India, Thailand, and Vietnam. Appl Environ Microbiol. 2015 May 1;81(9):3115-20. doi: 10.1128/AEM.00258-15

Müller, A., Stephan, R., & Nüesch-Inderbinen, M. (2016). Distribution of virulence factors in ESBL-producing Escherichia coli isolated from the environment, livestock, food and humans. *Science of the Total Environment*, *541*, 667–672. Doi:10.1016/j.scitotenv.2015.09.135

Araújo S, A T Silva I, Tacão M, Patinha C, Alves A, Henriques I. Characterization of antibiotic resistant and pathogenic Escherichia coli in irrigation water and vegetables in household farms. Int J Food Microbiol. 2017 Sep 18;257:192-200. doi: 10.1016/j.ijfoodmicro.2017.06.020.

Scheinberg JA, Dudley EG, Campbell J, Roberts B, DiMarzio M, DebRoy C, Cutter CN. Prevalence and Phylogenetic Characterization of Escherichia coli and Hygiene Indicator Bacteria Isolated from Leafy Green Produce, Beef, and Pork Obtained from Farmers' Markets in Pennsylvania. *J Food Prot.* 2017 Feb;80(2):237-244. doi: 10.4315/0362-028X.JFP-16-282.

Luo J, Yao X, Lv L, Doi Y, Huang X, Huang S, Liu JH. Emergence of mcr-1 in Raoultella ornithinolytica and Escherichia coli Isolates from Retail Vegetables in China. Antimicrob Agents Chemother. 2017 Sep 22;61(10):e01139-17. doi: 10.1128/AAC.01139-17.

Janalí­ková, M., Pleva, P., Pavlí­čková, S., Lecomte, M., Godillon, T., & Holko, I.(2018). Characterization of Escherichia coli strains isolated from raw vegetables. Potravinarstvo Slovak Journal of Food Sciences, 12(1), 304–312. Doi:10.5219/897

Ortega-Paredes D, Barba P, Mena-López S, Espinel N, Zurita J. Escherichia coli hyperepidemic clone ST410-A harboring blaCTX-M-15 isolated from fresh vegetables in a municipal market in Quito-Ecuador. Int J Food Microbiol. 2018 Sep 2;280:41-45. doi: 10.1016/j.ijfoodmicro.2018.04.037.

Liao N, Borges CA, Rubin J, Hu Y, Ramirez HA, Chen J, Zhou B, Zhang Y, Zhang R, Jiang J, Riley LW. Prevalence of β-Lactam Drug-Resistance Genes in Escherichia coli Contaminating Ready-to-Eat Lettuce. *Foodborne Pathog Dis*. 2020 Dec;17(12):739-742. doi: 10.1089/fpd.2020.2792.

Song J, Oh SS, Kim J, Shin J. Extended-spectrum β-lactamase-producing Escherichia coli isolated from raw vegetables in South Korea. Sci Rep. 2020 Nov 12;10(1):19721. doi: 10.1038/s41598-020-76890-w.

Chelaghma W, Loucif L, Bendahou M, Rolain JM. Vegetables and Fruit as a Reservoir of β-Lactam and Colistin-Resistant Gram-Negative Bacteria: A Review. *Microorganisms*. 2021 Dec 8;9(12):2534. doi: 10.3390/microorganisms9122534.

Oh SS, Song J, Kim J, Shin J. Increasing prevalence of multidrug-resistant mcr-1-positive Escherichia coli isolates from fresh vegetables and healthy food animals in South Korea. *Int J Infect Dis*. 2020 Mar;92:53-55. doi: 10.1016/j.ijid.2019.12.025

Massella E, Giacometti F, Bonilauri P, Reid CJ, Djordjevic SP, Merialdi G, Bacci C, Fiorentini L, Massi P, Bardasi L, Rubini S, Savini F, Serraino A, Piva S. Antimicrobial Resistance Profile and ExPEC Virulence Potential in Commensal Escherichia coli of Multiple Sources. *Antibiotics* (Basel). 2021 Mar 26;10(4):351. doi: 10.3390/antibiotics10040351.

Priyanka P, Meena PR, Raj D, Rana A, Dhanokar A, Duggirala KS, Singh AP. Urinary tract infection and sepsis causing potential of multidrug-resistant Extraintestinal pathogenic E. coli isolated from plant-origin foods. Int J Food Microbiol. 2023 Feb 2;386:110048. doi: 10.1016/j.ijfoodmicro.2022.110048.
